# The protective performance of reusable cloth face masks, disposable procedure masks, KN95 masks and N95 respirators: Filtration and total inward leakage

**DOI:** 10.1371/journal.pone.0258191

**Published:** 2021-10-06

**Authors:** Scott Duncan, Paul Bodurtha, Syed Naqvi

**Affiliations:** Defence Research and Development Canada - Suffield Research Centre, Chemical Threat Defence Section, Medicine Hat, Alberta, Canada; Valahia University of Targoviste, ROMANIA

## Abstract

Face coverings are a key component of preventive health measure strategies to mitigate the spread of respiratory illnesses. In this study five groups of masks were investigated that are of particular relevance to the SARS-CoV-2 pandemic: re-usable, fabric two-layer and multi-layer masks, disposable procedure/surgical masks, KN95 and N95 filtering facepiece respirators. Experimental work focussed on the particle penetration through mask materials as a function of particle diameter, and the total inward leakage protection performance of the mask system. Geometric mean fabric protection factors varied from 1.78 to 144.5 for the fabric two-layer and KN95 materials, corresponding to overall filtration efficiencies of 43.8% and 99.3% using a flow rate of 17 L/min, equivalent to a breathing expiration rate for a person in a sedentary or standing position conversing with another individual. Geometric mean total inward leakage protection factors for the 2-layer, multi-layer and procedure masks were <2.3, while 6.2 was achieved for the KN95 masks. The highest values were measured for the N95 group at 165.7. Mask performance is dominated by face seal leakage. Despite the additional filtering layers added to cloth masks, and the higher filtration efficiency of the materials used in disposable procedure and KN95 masks, the total inward leakage protection factor was only marginally improved. N95 FFRs were the only mask group investigated that provided not only high filtration efficiency but high total inward leakage protection, and remain the best option to protect individuals from exposure to aerosol in high risk settings. The Mask Quality Factor and total inward leakage performance are very useful to determine the best options for masking. However, it is highly recommended that testing is undertaken on prospective products, or guidance is sought from impartial authorities, to confirm they meet any implied standards.

## 1 Introduction

Face coverings (masks) are being used in many jurisdictions around the world as a preventative health measure (PHM) to mitigate the spread of the severe acute respiratory syndrome coronavirus 2 (SARS-CoV-2). On 11 March 2020 the World Health Organization (WHO) declared that a global pandemic was underway caused by SARS-CoV-2 [1].

The implementation of masking policies [2] is not without controversy. Some studies have made recommendations not to wear homemade face masks as a method of reducing transmission of infection from aerosols because of their limited effectiveness compared to proven filtering facepiece respirators, and potential misunderstanding of their performance, possibly promoting a false sense of safety [3, 4]. However, Worby *et al*. [5] makes the point that although face masks may have a limited protective effect, they can reduce total infections and deaths, and can delay the peak time of an epidemic. On purely physical grounds, mask use has merit. The literature is replete with examples showing that normal physiological respiratory activities such as sneezing, coughing, talking and even breathing, expel particles into the local environment where individuals socialise and work [6–11]. Related investigations have shown that the majority of particles and their evaporated nuclei generated through human expiration activities have sizes varying from 0.05 to 500 μm [12–16]. Particles in exhaled breath are typically <4 μm with a median 0.7 to 1.0 μm [17]. Dynamic respiratory events may project particulates well in excess of 2 m [18–21], which is also supported by physical modelling investigations [15, 22–25]. Particles of all sizes will be subject to evaporation leading to rapidly diminishing diameters and volumes [24, 26], and as diameters decrease, the settling time becomes longer [27]; from Stokes Law a 50 μm diameter particle will take ~20 s to settle 1.625 m in still air compared to ~41.6 h for a 0.5 μm diameter particle. The cut-off between aerosol and droplet may be an arguable point and it certainly lacks consensus. Various groups put it at 5 μm, 10 μm and 100 μm [28–30], but it is not particularly relevant in the context of potential inhalation exposure. There seems little doubt, when taking into account macro air movements related to heating and ventilation, convection, people activity, and even more localised radiative warming due to equipment, the human body and sunshine through windows, a large fraction of respiratory generated particles will be entrained and suspended in air currents and may potentially travel significant distances over time [31–34]. This may certainly add to the airborne viral load in situations where influenza (e.g. COVID-19) symptomatic individuals are shedding virus.

There is little direct evidence for the transmission of SARS-CoV-2 by any specific route [35]. Fennelly [36] comments that the logic that transmission within close proximity presupposes respiratory disease spread by droplets is fallacious, and he correctly points out that small particle aerosol are typically in the highest concentration in close interactions with others. Several research groups have demonstrated that SARS-CoV-2 remains viable in aerosol 1 to 3 μm diameter for periods of ~3 to 16 hours [37, 38]. With the progression of the pandemic there is a growing body of literature that supports airborne transmission of SARS-CoV-2 playing an increasingly important role in the spread of the disease [29, 39–44]. Numerous earlier studies have established that aerosol expelled during normal respiratory activities can contain infectious influenza virus [42, 45–54]. It stands to reason that covering one’s mouth and nose with a face covering of any type will, at the very least, reduce the number of particulates expelled by the wearer and lessen the exposure to particulates produced by others. The construct that masks play a dual role, protecting the external environment from an individual and the individual from the external environment, has been discussed previously [45, 55, 56]. However, to be wholly effective and provide wearers and others an appropriate level of protection against an aerosol hazard, a mask must restrict particulates from entering the inside cavity next to the face, and filter particulates from both the inhalational and exhalational air stream. To achieve improved mask performance will depend significantly on the face covering design, in particular the face seal, and incorporating materials used in their construction that exploit all mechanisms of filtration [57].

Masks that have been in use during the SARS-CoV-2 pandemic can be broadly categorized into five groups; i) re-purposed apparel such as scarfs, neck warmers, bandanas, etc., ii) simple fabric masks of one or two layers, iii) fabric masks with an included layer specifically to augment filtration efficiency, iv) disposable procedure/surgical masks of the kind used in health care settings, and v) certified filtering facepiece respirators (FFR), such as FFP2, KN95 and N95 masks. N95 FFR are employed as personal protective equipment (PPE) and they provide a high level of protection for filtering facepiece masks that cover only the nose and mouth (as opposed to full and half-face respirators constructed from elastomeric materials) [3, 58–74]. N95 FFRs are certified under the National Institute for Occupational Safety and Health (NIOSH) [75]. They must limit the mass penetration of non-oil-based particulates to <5%, and they must provide a fit factor (FF) ≥100 when quantitatively fit to individuals; a quantitative fit test (QNFT) only measures inward leakage at the face seal. Measured protection factors (PFs) of N95 FFRs when worn in the workplace have been shown to give considerably lower protection [58, 59], and both the Canadian Standards Association (CSA) and the US Occupational Safety and Health Association (OSHA) have given these respirators an assigned protection factor (APF) of 10 [76, 77]. For disposable procedure/surgical masks and reusable fabric face coverings, there is no requirement to provide a specific level of protection performance, whether for filtration efficiency or inward leakage. Although just recently, new protection standards for barrier face coverings (BFCs) have been developed by the American Society of Testing Material (ASTM) [78]. The standard was primarily established in response to the global COVID-19 pandemic to address a product that is neither a medical face mask per ASTM Specification F2100 [79] for providing source control, nor a respirator for providing inhalation protection as defined by regulatory requirements specified in the United States under 42 CFR Part 84 [80]. This specification is intended to establish a US national standard for a BFC, identifying how the device should perform in terms of source control/protection, comfort, and re-use potential.

The ability of materials used in homemade face coverings to remove particulates is completely dependent on the characteristics of the fabric, such as the weave type and fiber structure [4, 62, 81], as well as number of fabric layers. Materials used in disposable procedure/surgical masks afford better filtration than homemade masks but typically are substantially less efficient than N95 FFR materials [45, 56, 68]. In comparison to N95 FFRs, and given the absence of any type of face seal, masks made from common apparel fabrics and procedure/surgical masks afford only minimal protection levels. Inward leakage due to gaps in the fit has been demonstrated to play a critical role in the ability of a mask or respirator to protect against exposure to particulates [4, 59, 82–84]. To our knowledge, there have been two studies that have measured the total inward leakage on a variety of surgical and procedure masks [55, 59], and currently only one study that has measured the TIL for home-made masks made of cloth materials with testing performed on only one volunteer [85]. The present study provides a more comprehensive, side-by-side assessment of mask material filtration performance and the TIL of particulates into masks when worn by well-trained individuals.

In this study we investigate five groups of masks of particular relevance to the SARS-CoV-2 pandemic: N95 FFRs, KN95 masks, disposable procedure/surgical masks, and reusable, fabric two-layer and multi-layer masks. Filtration testing of mask materials is normally performed at standard test flow rates of either 28 L/min, to match the flow rate used in ASTM filtration test methods [78, 86, 87], or 85 L/min, to match the flow rate used by NIOSH for N95 respirator filtration testing [80]. However, these flow rates are significantly higher than a representative breathing expiration rate for a person in a sedentary or standing position conversing with another individual (i.e., 17 L/min) [88]. We have assessed the filtration efficiency of the mask material at this lower flow rate, and to the best of our knowledge no other paper has performed filtration testing at this representative breathing expiration rate. It is reasonable to assume that penetrated particle distributions containing larger particles are likely to carry more virus. Importantly, notwithstanding differences in viral load concentration, an inhalational exposure to any number of virions will be heavily dependent on the particle penetration profile of the mask and the shape and size range of the particle distribution. We demonstrate that there is a dramatic range in the potential for exposure to virus entrained in aerosol depending on the filtration efficiency of the mask material, the aerosol challenge distribution and the viral load concentration. Total inward leakage can play an important role in the ability of face coverings to reduce exposure to aerosol, and may in fact be more imperative than the efficiency of the filtering material used in a mask. To gain a better understanding of this relationship, the total inward leakage protection performance of the five face covering groups, worn by a cross-section of both males and females with a wide distribution of facial sizes, was also investigated in this study. This affords a direct comparison between face covering protection performance and filtration efficiency for key categories of masks of importance to the broader mask user community, including the general public.

## 2 Materials and methods

### 2.1 Test items

**Fig 1:**
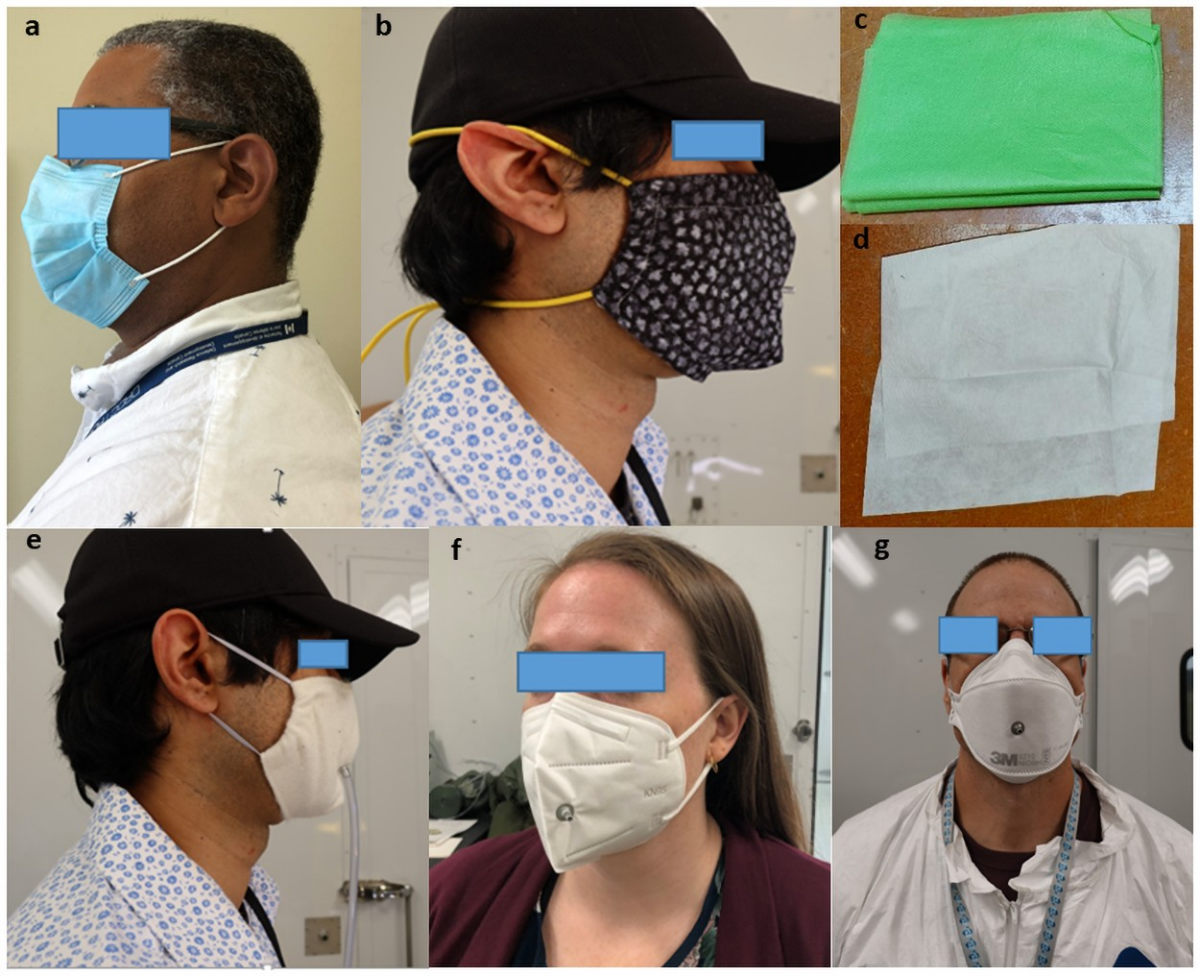
(a) Disposable procedure mask: Henan Liwei Biological Pharmaceutical Co. Ltd; (b) Multi-layer mask: four layered system comprised of two layers of quilt batting between an outer and inner layer of cotton; (c) Non-woven polypropylene ‘craft’ material (manufactured by smart-fab^®^); (d) “N95-like” electret filter membrane material (purchased from Amazon.ca) used as the middle filter layer, as part of a multi-layer mask; (e) 2-Layer mask: quilt batting/cotton mask; (f) KN95 mask: MedSup Canada Protective Face Mask, and; (g) N95 FFR: 3M^™^ model 9210.

This study investigated five groups of masks of particular relevance to the SARS-CoV-2 pandemic: N95 FFRs, KN95 masks, disposable procedure/surgical masks, and reusable, fabric 2- and multi-layer masks. Each mask group included variants similar in style and function: for example, the multi-layer mask group consisted of masks constructed from an inner (next to skin) fabric layer, an outer (facing the environment) fabric layer and a middle layer specifically to augment the filtration efficiency of the material system. Fig 1 shows select masks from the fives groups tested. Additional photographs are provided in S1 Appendix, including a table (S9 Table in S1 Appendix) that lists the make/model and manufacturer, source of all the mask materials tested, and the type of test (filtration efficiency (FE), total inward leakage (TIL) and/or QNFT) performed on each type of mask material used in the study.

#### 2.1.1 Fabric 2-layer and multi-layer masks

The fabric 2-layer mask group included five variations constructed from quilt batting, cotton, nylon, polyester and silk. The multi-layer mask group included seven variants all constructed with a cotton inner and outer layer but different middle filter layer. The latter included: furnace filter (3M^™^ Filtrete furnace filter, Merv 13), quilt batting, electret filter membrane, disposable procedure mask, non-woven polypropylene shopping bag material and two types of non-woven polypropylene craft material (smart-fab^®^).

#### 2.1.2 Procedure/Surgical masks

The procedure/surgical mask group included six disposable face coverings of the type typically used in hospital/clinical settings and many commercial establishments; Model PG4-1200 PrimaGard (PG4-1200), Model PG4-1273 PrimaGard Level 3 Barrier (PG4-1273), Model PG4-2001 PrimaGard Surgical Mask Level 1 Barrier, Tie, (PG4-2001) and PrimaGard Level 1 Barrier (PG4-2331), all obtained from priMED Medical Products Inc. (Edmonton, Canada); Model 836185 Disposable Face Mask (Henan Liwei), Henan Liwei Biological Pharmaceutical Co. Ltd, and; Vanch Disposable Medical Face Mask (V-DMFM), 2020 Beifa Group Co. Ltd., Ningbo China.

#### 2.1.3 KN95 masks and N95 filtering facepiece respirators

The KN95 mask group includes two models: MedSup Canada KN95 Protective Face Mask (MedSup Canada) and the TAIDAKANG KN95 Protective mask (TAIDAKANG). Both masks were certified to the Chinese standard GB2626-2006 (equivalent to a FE ≥ 95% and a TIL ≤ 8%) [89].

The N95 FFR group included seven models: 3M^™^ model 1870 (fold style); Halyard Health, model FLUIDSHIELD 2 N95 (duck bill fold style); North Safety Products model 7130N95 (cup style); Gerson model 2130 N95 Respirator, Louis M. Gerson Co., (rectangular cup style); 3M^™^ model 8110s (cup style, size small); 3M^™^ model 9210 (fold style). All masks were certified to the US NIOSH FFR standard [80]. The FFRs were chosen specifically for their differences in form and manufacturer, providing variability in respirator fit and filtration.

### 2.2 Evaluation of aerosol penetration through mask fabrics

**Fig 2:**
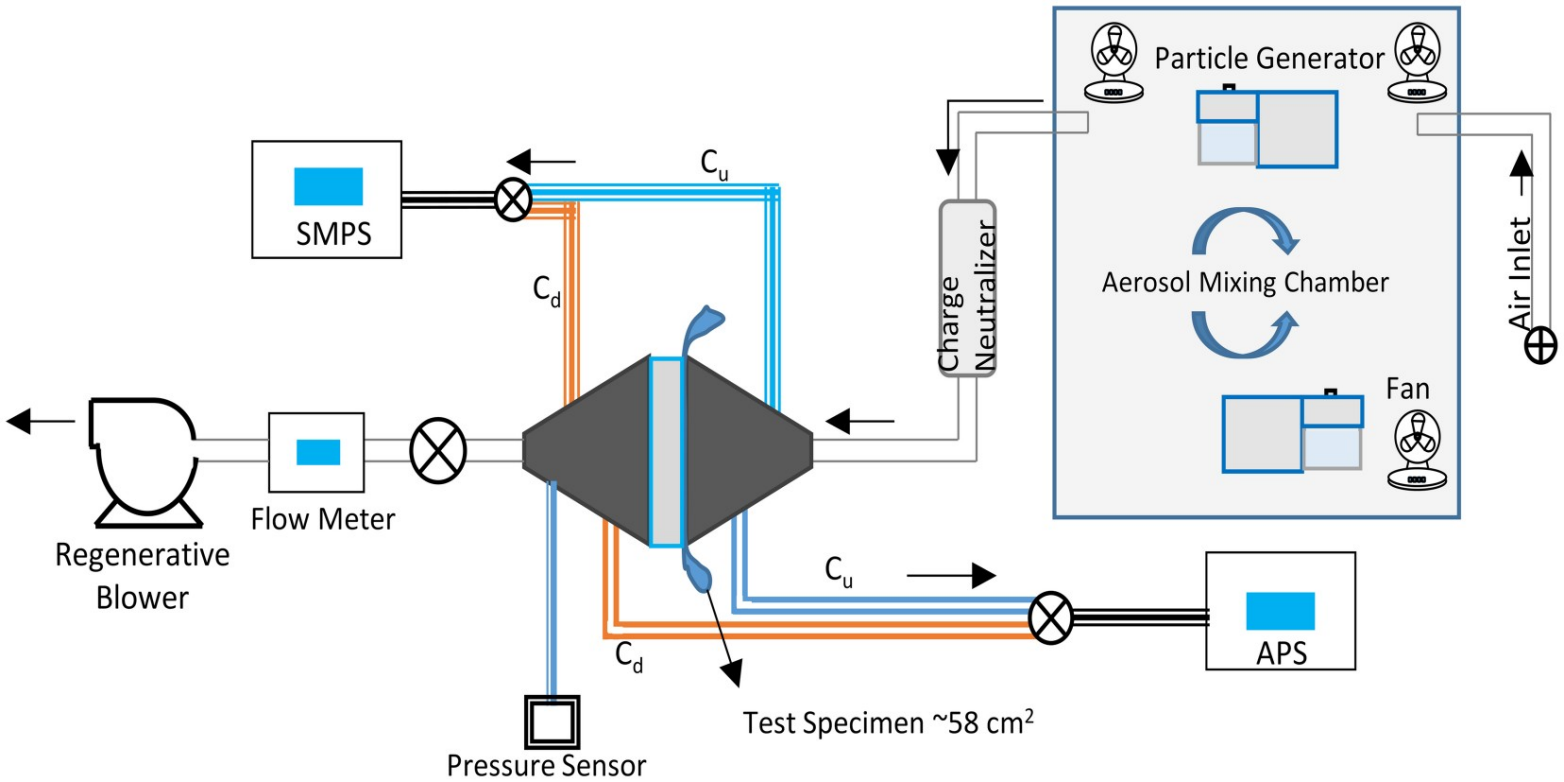
Schematic of aerosol swatch penetration set-up. A polydisperse NaCl aerosol is generated in the mixing chamber and pulled through the charge neutralizer and swatch test rig using a regenerative blower. Air flow is regulated using the flow meter. Aerosol concentration, upstream and downstream, is measured with the SMPS/APS as shown.

The experimental aerosol swatch penetration set-up is shown in Fig 2. Located within the mixing chamber are two aerosol generators (TSI Model 8026, Shoreview, USA) and three fans positioned to maintain a steady uniform aerosol concentration of inert sodium chloride (NaCl) particles between 45,000–60,000 particles/cm^3^, permitting a reliable measurement of filtration efficiency of greater than 99.99%. A scanning mobility particle sizer (SMPS) spectrometer (TSI model 3080 with long differential mobility analyzer TSI model 3081 and ultrafine condensation particle counter TSI model 3776) was used to measure aerosol over a mobility particle size range 0.023–0.67 μm, and an aerodynamic particle sizer (APS) spectrometer (TSI model 3321) was used to measure aerosol over an aerodynamic size range 0.5–5 μm. The aerosol distribution in the mixing chamber was polydisperse, extending over the range from ~0.023 μm to ~5 μm, with a count median mobility particle diameter of 0.076 μm (geometric standard deviation of 1.86) corresponding to a count median aerodynamic particle diameter of 0.131 μm (geometric standard deviation 1.75), respectively. The use of NaCl particles as a challenge aerosol, and its associated size distribution, has been shown to be an appropriate simulant to represent size ranges of both bacteria and viruses [3, 59]. Balazy *et al*. [90] has shown similar penetration of MS2 virus and sodium chloride particles through N95 FFRs and surgical masks. Moreover, the use of NaCl aerosol is an accepted challenge medium for filtration testing of N95 FFRs by [75] to quantify and qualify the performance of respirators.

The aerosol size distribution, concentration, relative humidity (RH) and temperature ((25 ± 5°C and 30 ± 10% RH) were measured to be in compliance with US Code of Federal Regulations [80] as referenced by [75], and ASTM [78] for filtration testing of N95 FFRs and BFCs, respectively. Aerosol from the mixing chamber is drawn through a charge neutralizer (TSI Model 3054) in compliance with US CFR [80], neutralizing the surface charge on the particles to a Boltzmann equilibrium state, and then drawn through the fabric in the sample holder. A regenerative blower (GAST model R1102K-01), needle valve and digital flow meter (TSI Model 5330–2) were used to adjust the flow rate and a t-valve to adjust the concentration between upstream and downstream. The fabric sample holder consisted of a central housing in two parts joined by a clamp to hold the material sample in place with no leaks. The holder allowed for an exposed sample area of 58.1 cm^2^. Face coverings were tested at a flow rate of 17 L/min to simulate an expiratory flow rate for a person at rest with a minute volume between 9 and 11 L/min (inspiration to expiration ratio of 1:1.5) [88, 91]. The corresponding face velocity was 4.87 cm/s. The N95 FFRs, due to their variable three dimensional form, had face velocities from 1.10 to 6.69 cm/s. The three dimensional form of the KN95 masks resulted in a face velocity of 2.01 cm/s. The inhalation pressure drop (ΔP) across the face covering materials was measured with an Ashcroft CXLdp Pressure transducer (Part# CX4MB21015IWL-XRH and CX4MB210P25IWL-XRH) to determine inhalation breathing resistance.

#### 2.2.1 Calculation of aerosol penetration through mask fabrics

The aerosol penetration through the face covering materials was determined by measuring the aerosol concentration upstream and downstream of the material in the sample holder, and calculated according to the following formula

$$P_{e}(\%)=1-FE=(1-\frac{\left(C_{u}-C_{d}\right)}{C_{u}})\times 100$$ (1)

where *P*_*e*_, *C*_*u*_ and *C*_*d*_ are the penetration, concentration upstream and concentration downstream per particle bin, respectively, and *FE* is the filtration efficiency. Measurement of the upstream and downstream concentrations was accomplished through a series of manually operated valves to direct the upstream and downstream flow through the SMPS and APS as required. Up to four upstream and downstream measurements were repeated on each mask material. An example of the variability for repeat measurements, adjusting for small changes in the challenge concentration, is provided in S1 Fig in S1 Appendix. Mobility particle diameter data obtained from the SMPS was converted to aerodynamic diameter and combined with the APS aerodynamic particle size data. Aerodynamic diameter finds wide application in aerosol technology as it standardizes a particle on shape (a sphere) and density (1g/cm^3^). The conversion of mobility to aerodynamic diameter is referenced by Baron *et al*. [92], with the equation provided in S1 Appendix. All particle diameters referenced in our study will be in aerodynamic diameter. In the region where the SMPS and APS aerodynamic particle diameter overlapped (0.582 μm to 0.682 μm), a one to one average was used. The mean representative penetration for each mask group was determined from the ratio of the average of the downstream measurements to the average of the upstream measurements. The final composite aerodynamic diameter distribution reported here extends over the size range 0.027 μm to 5.0 μm, as very few particles >5 μm were measured. For some tests, the maximum upper range of the particle size distribution was ~3.5 μm, as there was not always sufficient concentration of larger particles to reliably measure penetration of higher particle size bins.

#### 2.2.2 Calculation of mask fabric protection factor

For all mask materials within each mask group we determine an overall, harmonic mean fabric protection factor (FPF) value by first taking the reciprocal of the mean number penetration for each particle diameter in the distribution, converting to a PF, and then calculate a harmonic mean FPF according to

$${FPF}_{HM}=(\frac{n}{\sum_{i=1}^{n}\frac{1}{{PF}_{i}}})$$ (2)

where ${PF}_{i}=P_{e_{i}}^{-1}$, *P*_*e*_ is the mean number penetration, *i* is the specific particle bin and *n* the total number of bins spanning the particle distribution measurement. The harmonic mean FPF is a very useful approach to describe the overall protection performance of a fabric against the entire particle distribution. It gives greater emphasis on the contribution of lower PF values in a sample group, more so than large PF values, particularly larger outliers. It is routinely used by the US OSHA, NIOSH and CSA for calculating overall protection and fit factors in respiratory protection standards [75, 76, 93, 94]. A geometric mean FPF was then calculated from the FPF measurements of all the masks materials within each mask group.

### 2.3 Evaluation of mask total inward leakage

**Fig 3:**
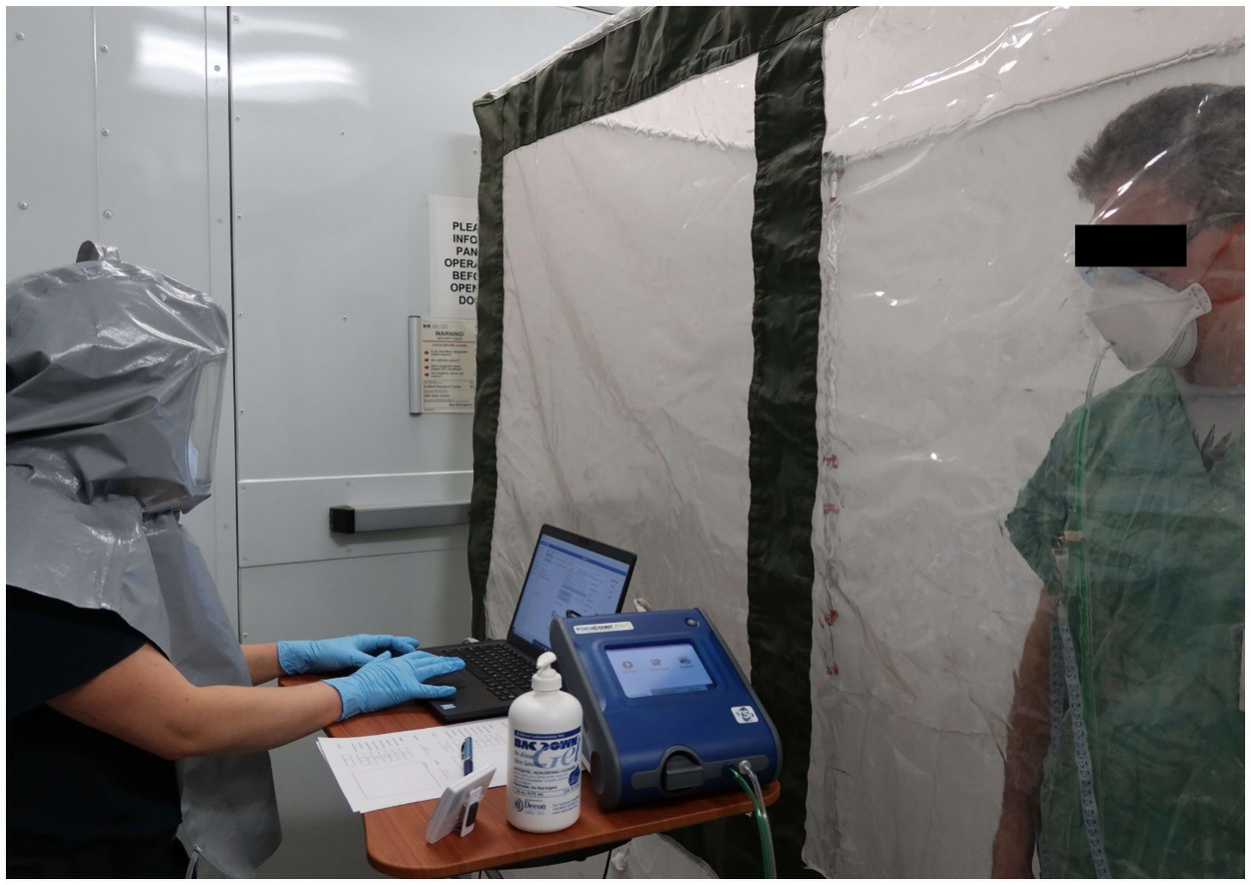
Total inward leakage test being performed on the 3M^™^ model 9210 N95 FFR. The Sampling probe for the TSI Portacount 8038 is shown to be connected to the respirator by use of a sampling probe supplied by TSI. The operator is shown to be wearing a loose-fitted PAPR and nitrile gloves as a health and safety precaution from working in close proximity to the test volunteers during the COVID-19 pandemic.

This test measured the total inward leakage (TIL) of aerosol into the facial cavity of a mask worn by a test subject. It represents the combined penetration of aerosol though gaps where the mask contacts the face, as well as the fraction that penetrates through the filtering material used in the construction of the mask. The same aerosol particle size distribution and concentration was used for the TIL mask measurements as was used for the aerosol penetration swatch test. Our TIL test incorporated seven activities involving head/face/body movements, each 30 s in duration, as recommended by the CSA for a quantitative fit test [76]. The TIL measurements were obtained with the TSI PortaCount model 8038 and using custom software (Royal Military College of Canada, Kingston). To measure the aerosol concentration inside the face covering/FFR a rivet-style sampling probe (TSI Model 8025-N95Adaptor Kit) was inserted through the material at a location between the mouth and nose of the subject and connected to the PortaCount via tubing. The external aerosol was sampled from a separate line connected to the instrument approximately 10 cm in front of the individuals face. Fig 3 shows the setup for performing the total inward leakage measurement.

A pool of eleven subjects were recruited for this test from Defence Research and Development Canada (DRDC) Suffield Research Centre (Ralston, Canada) and were chosen based on obtaining a cross-section of both males and females, with a wide distribution of age, height and weight. Based on the bivariate panel described by [95] for facial size distribution, five subjects were of smaller facial sizes, four were of medium facial sizes, and two were larger facial sizes. All subjects were knowledgeable, trained face covering/FRR users, and were successfully quantitatively fit tested to FFRs (fit factor >100) using the TSI PortaCount model 8038 according to CSA [76]. This study was undertaken internal to Defence Research and Development Canada (DRDC), an agency within the Canadian Department of National Defence. Only the scientific research employees of this organization were involved in the study. As such, it was deemed an internal DRDC research project and organizational policy did not require an ethics review. Informed consent was obtained for the total inward leakage tests.

## 3 Results

### 3.1 Particulate penetration through mask fabrics

Values for the Fabric Protection Factor, Quality Factor, face velocity, inhalation pressure drop, and number penetration as a function of aerodynamic particle diameter, for each mask tested in all five mask groups in this study are provided in S1 Table and S2-S6 Figs in S1 Appendix.

#### 3.1.1 Fabric 2-layer masks

**Fig 4:**
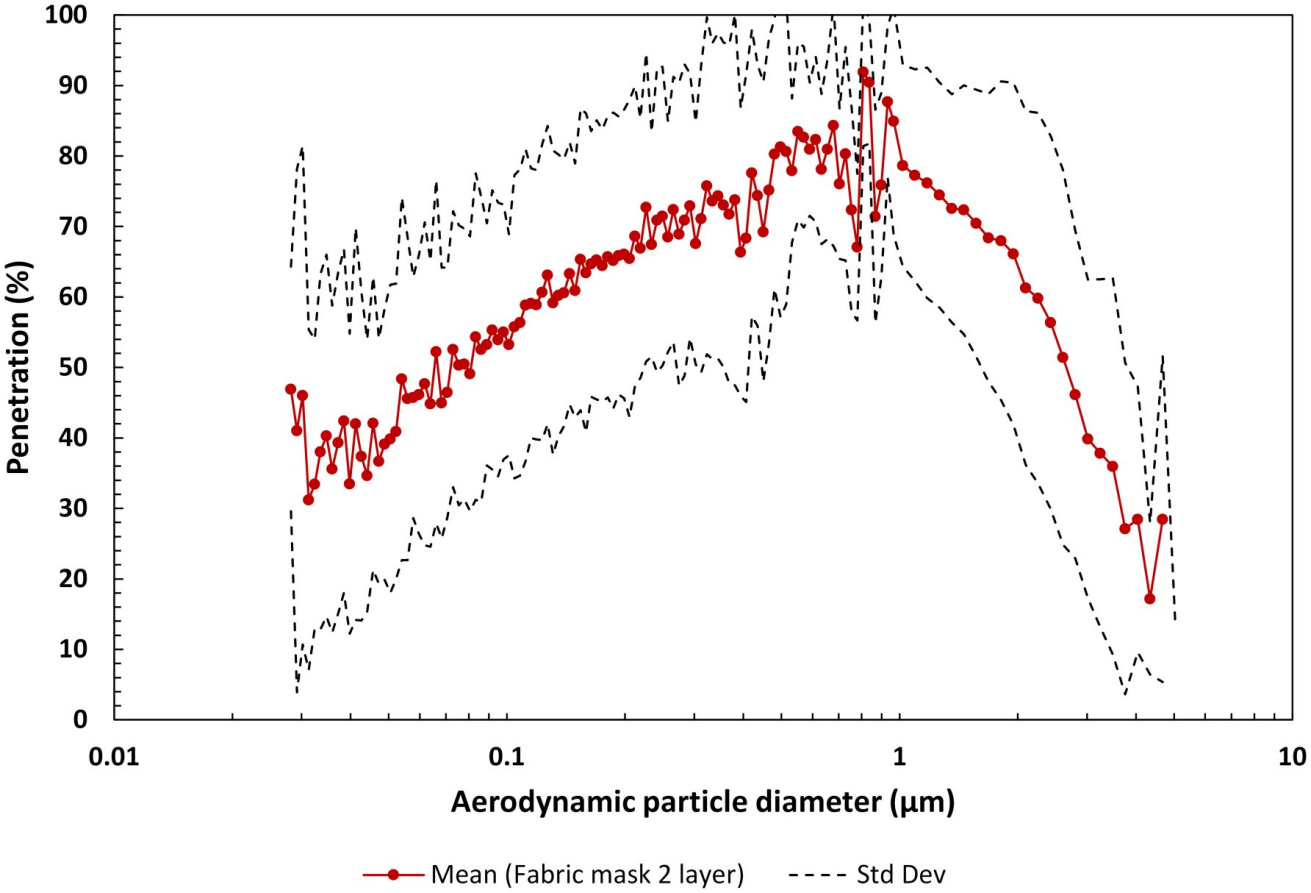
Mean penetration profile as a function of aerodynamic particle diameter for the material of five fabric masks comprised of 2 layers. Note, the penetration range is 0 to 100%. Materials included quilt batting, cotton, nylon, polyester and silk.

Many different fabrics are being considered for reusable face coverings. Common apparel materials that individuals may have available include cotton, nylon, polyester, silk, and quilt batting. Fig 4 presents penetration, based on mean number count, as a function of aerodynamic particle diameter for five fabrics, each consisting of two layers. Additional detail is provided in Table 1. The maximum mean penetration for the five fabrics was 91.9% at a particle size of 0.809 μm. The geometric mean FPF for the fabric 2-layer group was determined to be 1.78 with a geometric standard deviation (GSdev) of 1.22, corresponding to an overall particle penetration for these materials of ~56%. Inhalation pressure drop across the fabrics depended on the tightness of the weave (air permeability) and ranged from 22.6 Pa for two layers of quilt batting to 375.7 Pa for two layers of nylon. Face velocity was 4.87 cm/s.

**Table 1:** Maximum penetration, geometric mean FPF, percent overall penetration, inhalation pressure drop and face velocity for the five mask groups: Fabric 2-layer, multi-layer, disposable procedure, KN95 and N95 FFRs.

Mask Type	% Max penetration (MPPS μm)	Geomean FPF (GSdev)	% Overall Penetration	Inhalation pressure drop (Pa)	Face velocity (cm/s)
Fabric 2-layer masks	91.9 (0.809)	1.78 (1.22)	~56	22.6 to 375.7	4.87
Multi-layer masks	45.2 (0.37)	3.61 (1.57)	~28	31.4 to 60.8	4.87
Disposable procedure masks	26.4 (0.058)	9.73 (1.17)	~10	21.6 to 50.0	4.87
KN95 masks	2.28 (0.123)	144.5 (1.71)	~0.7	37.3 to 67.7	2.01
N95 FFRs	3.41 (0.076)	69.8 (2.23)	~1.4	45.5 to 56.4	1.10 to 6.69

MMPS = Maximum penetrating particle size; Geomean = geometric mean; GSdev = geometric standard deviation.

#### 3.1.2 Multi-layer masks

**Fig 5:**
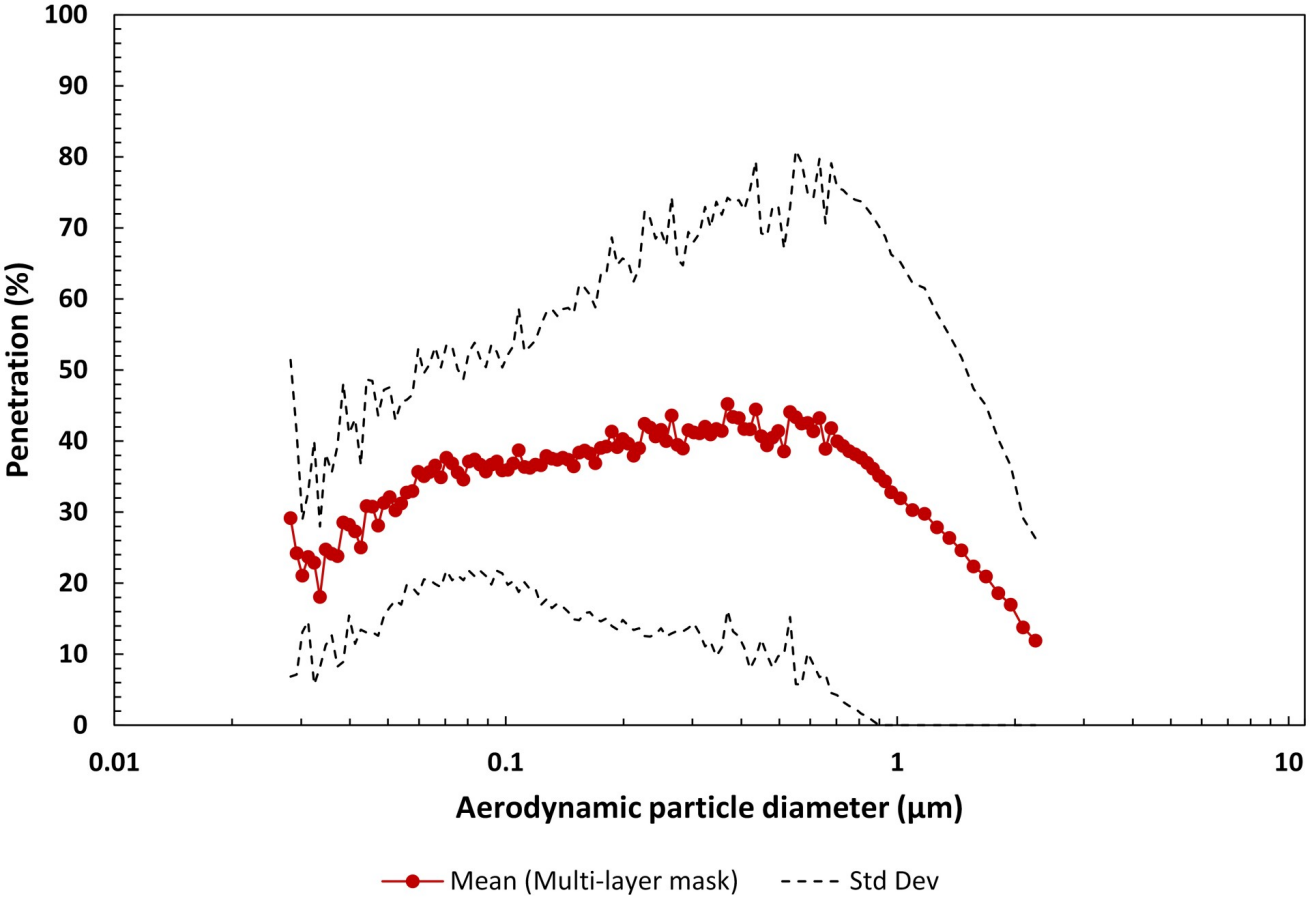
Mean penetration profile as a function of aerodynamic particle diameter for the material of seven fabric masks with an internal layer to augment filtration. Note, the penetration range is 0 to 100%. Multi-layer material systems included: i) cotton/electret furnace filter/cotton, ii) cotton/procedure mask/cotton, iii) cotton/electret filter membrane/cotton, iv) cotton/quilt batting/cotton, v) cotton/polypropylene shopping bag/cotton, vi) cotton/polypropylene craft 1/cotton, and vii) cotton/polypropylene craft 2/cotton.

Aerosol penetration was measured through reusable, multi-layer masks incorporating an internal layer specifically to augment their overall filtration efficiency. Material systems included: cotton/electret furnace filter/cotton, cotton/procedure mask/cotton, cotton/electret filter membrane/cotton, cotton/quilt batting/cotton, cotton/polypropylene shopping bag/cotton, cotton/polypropylene craft 1/cotton and cotton/polypropylene craft 2/cotton. The addition of the third layer was evaluated solely for its impact on mask filtration efficiency, and further consideration of other physical/chemical properties may be warranted for general mask use. The maximum mean penetration for this group was 45.2% at a particle size of 0.37 μm. The geometric mean FPF for the multi-layer group was determined to be 3.61 (GSdev 1.57), corresponding to an overall particle penetration for these materials of ~28% (Fig 5 and Table 1). Notably, the penetration through the multi-layer systems that included polypropylene filtering material was markedly higher than the other four systems investigated (see S3 Fig in S1 Appendix). Excluding the polypropylene filtering material systems the FPF was higher, 6.25 (GSdev 1.18), resulting in a lower overall particle penetration of ~16%. Inhalation pressure drop across the multi-layer fabric systems ranged from 31.4 Pa for the cotton/polypropylene craft 2/cotton to 60.8 Pa for the cotton/procedure mask/cotton system. Face velocity was 4.87 cm/s.

#### 3.1.3 Disposable procedure/surgical masks

**Fig 6:**
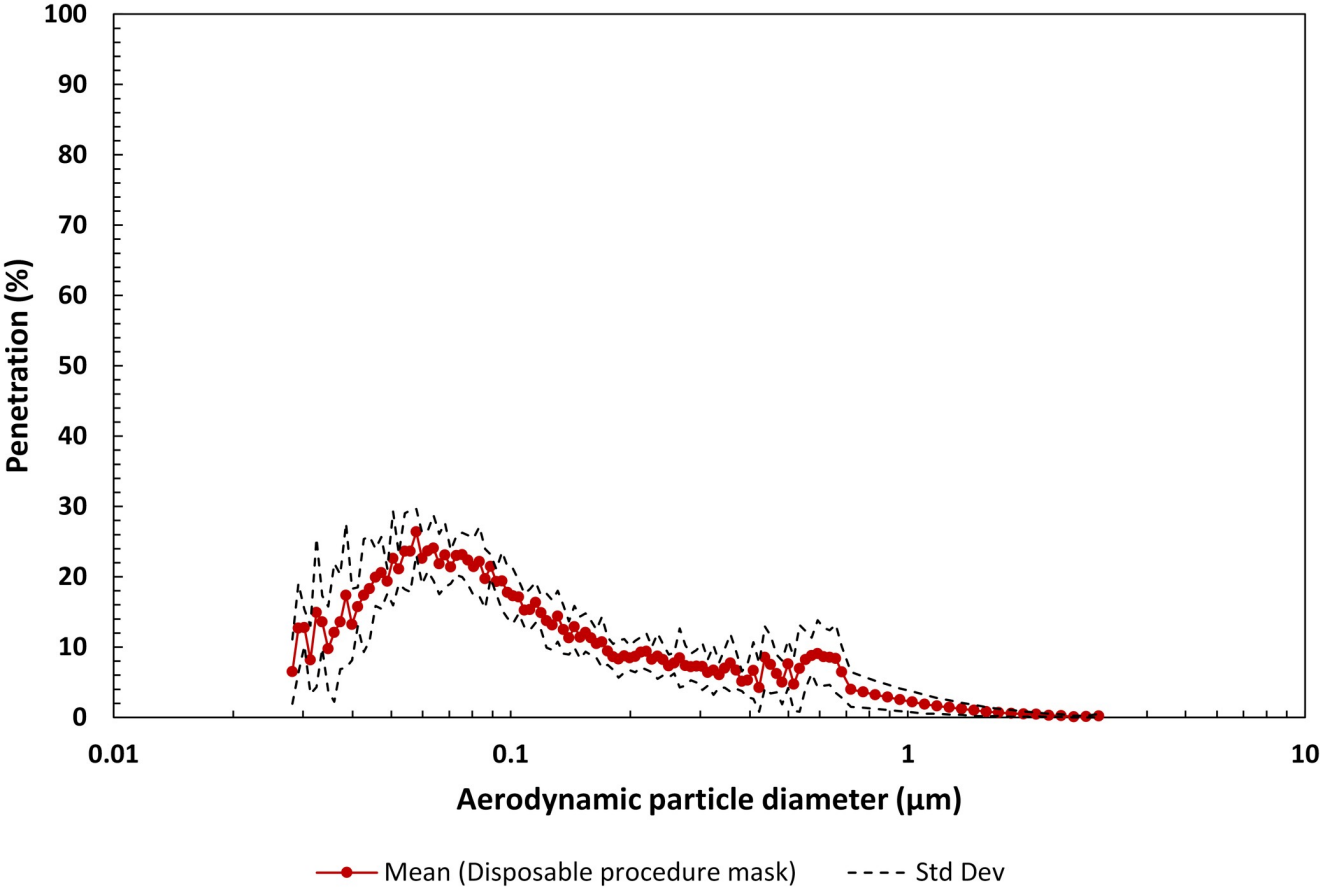
Mean penetration profile as a function of aerodynamic particle diameter for the filter material of six disposable procedure masks. Note, the penetration range is 0 to 100%. The bump in the penetration data between the particle range 0.5 to 0.67 μm is related to averaging the SMPS and APS measurements.

There are many manufacturers of disposable procedure masks and most manufacturers offer numerous models. In this study we investigated disposable procedure/surgical masks distributed/manufactured by three different companies as well as three additional models from one of the companies, for a total of six disposable procedure masks. The maximum mean penetration was 26.4% at a particle size of 0.058 μm (Fig 6). The geometric mean FPF for the disposable procedure/surgical mask group was determined to be 9.73 (GSdev 1.17), corresponding to an overall particle penetration for these materials of 10.3%. Inhalation pressure drop across the procedure masks ranged from 21.6 Pa to 50.0 Pa. Face velocity was 4.87 cm/s.

#### 3.1.4 KN95 mask

**Fig 7:**
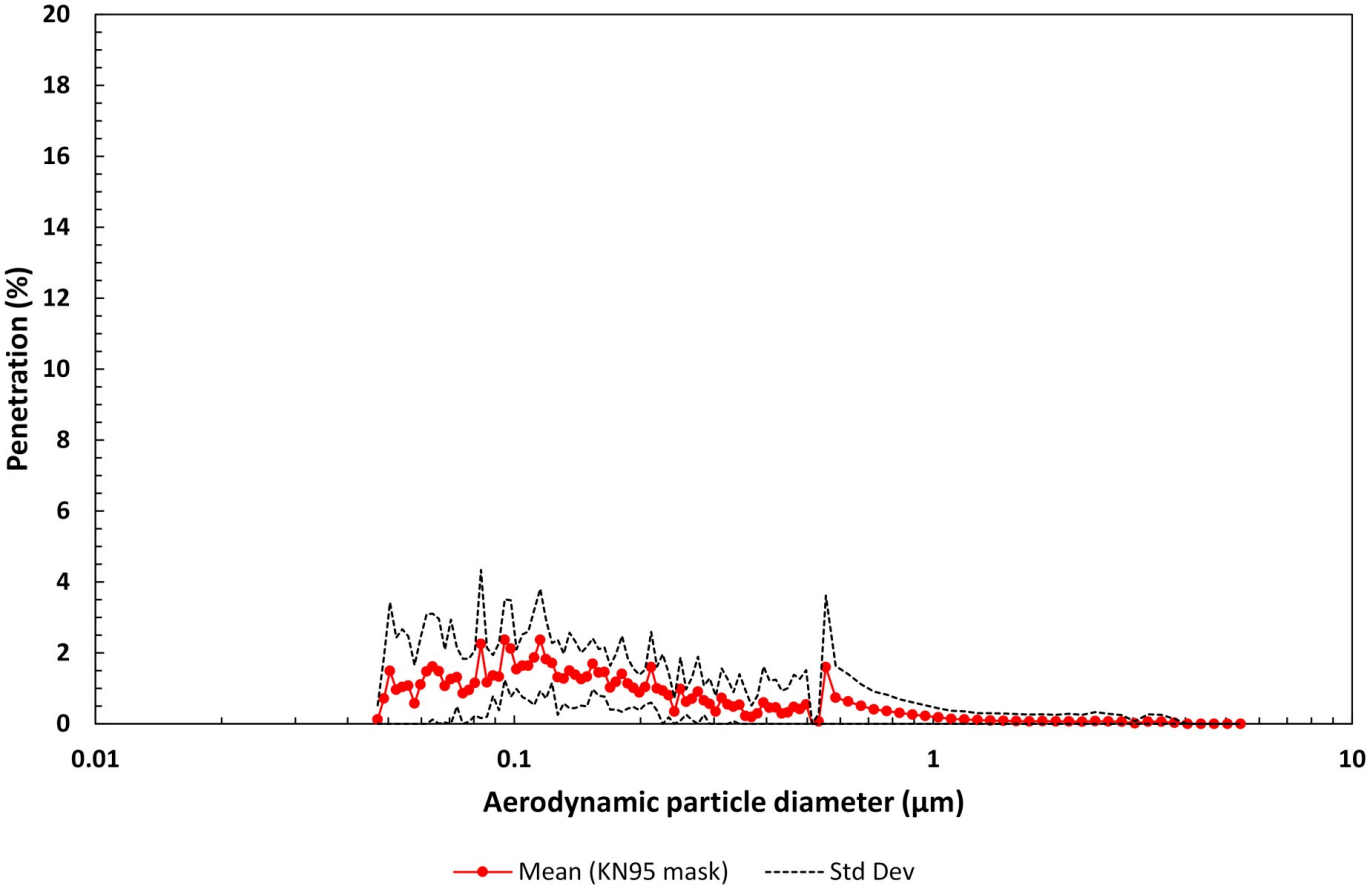
Mean penetration profile as a function of aerodynamic particle diameter for the filter material of two KN95 masks (five replicates each). Note, the penetration range is 0 to 20%.

Similar to procedure masks, there are numerous types of KN95 masks available. We investigated two of the more common brands. KN95 masks have not been designed to meet NIOSH N95 FFR certification [75] requirements: they are typically certified to a Chinese standard (i.e., country of origin) and have comparable filtration efficiency to N95 FFRs but a total inward leakage requirement of ≤ 8%, equivalent to a FF of ≤ 12.5 [89]. The maximum mean penetration for this group was 2.28% at a particle size of 0.123 μm. The geometric mean FPF for the KN95 mask group was determined to be 144.5 (GSdev 1.71), corresponding to an overall particle penetration for these materials of ~0.70% (Fig 7 and Table 1). Inhalation pressure drop for the two KN95 mask materials varied from 37.3 Pa to 67.7 Pa. Face velocity was 2.01 cm/s.

#### 3.1.5 N95 FFR

**Fig 8:**
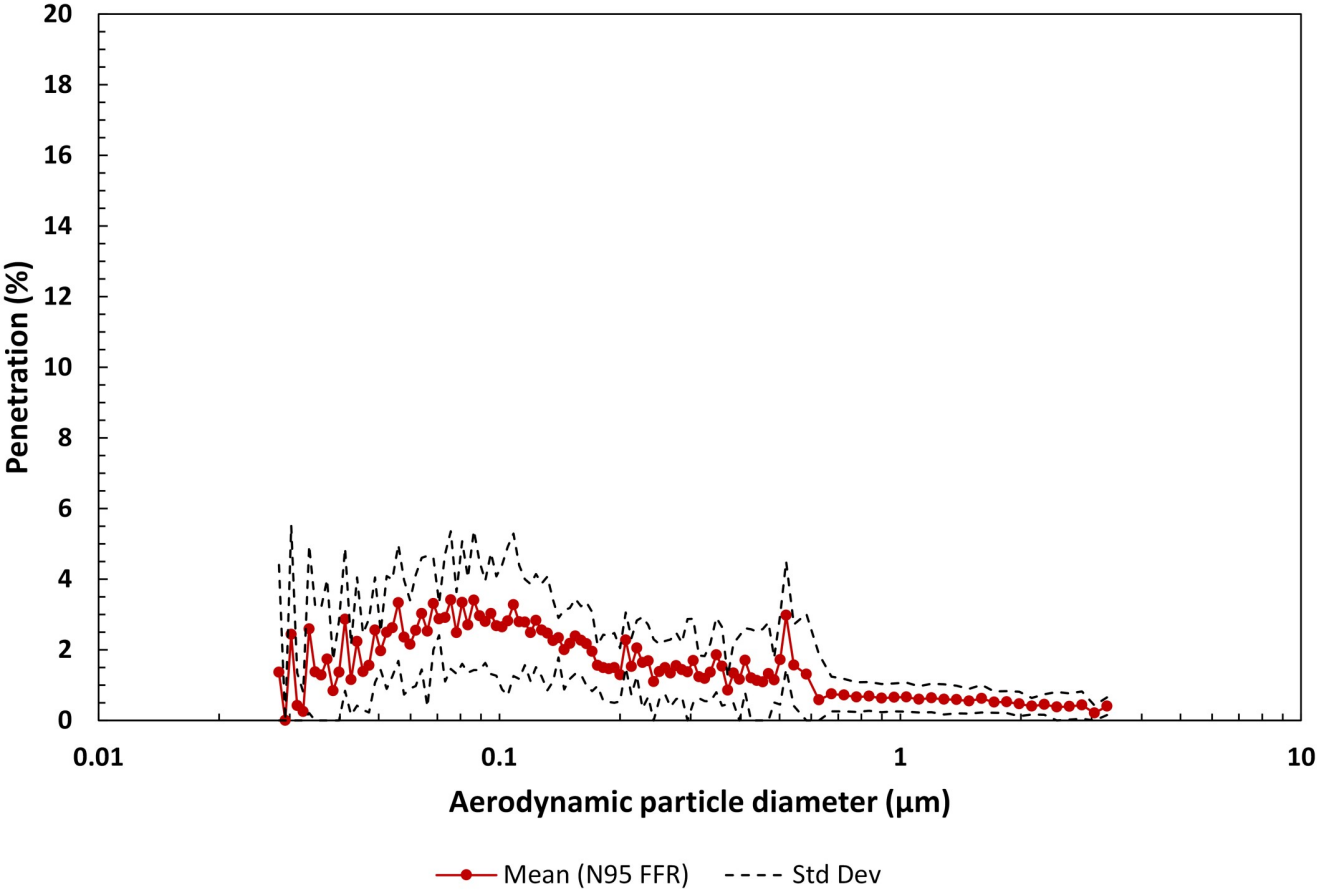
Mean penetration profile as a function of aerodynamic particle diameter for the filter material of five N95 FFRs (three replicates each). Note, the penetration range is 0 to 20%.

The filtration performance of any filtering material for use in a N95 FFR must meet the requirements of US CFR [80], as referenced by NIOSH [75], and the mass penetration of non-oil-based particulates cannot exceed 5% at a flow rate of 85 L/min. All N95 FFRs in our study met this requirement. The mean number count penetration as a function of aerodynamic particle diameter for five N95 FFR filtering materials is shown in Fig 8. Our results are similar to other filtration studies that have used a SMPS to measure aerosol penetration [60]. The mean maximum penetration for the five N95 FFRs was 3.41% at a particle size of 0.076 μm. The geometric mean FPF for the N95 FFR group was determined to be 69.8 (GSdev 2.23), corresponding to an overall particle penetration for these materials of ~1.4%. Inhalation pressure drop and face velocity ranged from 45.5 to 56.4 Pa and 1.10 to 6.69 cm/s respectively. As expected, these values are less than when N95 FFRs are tested at 85 L/min according to N95 FFR filtration testing standards [80].

### 3.2 Mask total inward leakage performance

Total inward leakage protection level tests were completed on a range of representative masks for each mask group. Fabric 2-layer masks included those made from cotton (two variants, eight replicates each), silk (five replicates), and quilt batting/cotton (five replicates). Multi-layer masks included those made from cotton/electret furnace filter/cotton (five replicates), cotton/quilt batting/cotton (five replicates), cotton/PM 2.5 filter/cotton (five replicates) and a nylon multi-laminate (five replicates). The latter two multi-layer mask types were included because they afforded filtration efficiency on par with filtering materials used in N95 FFRs, but had much higher inhalation pressure drops (110 Pa and 261 Pa respectively). Masks with the polypropylene filtering material were not made and evaluated because the filtration efficiency of these multi-layer material systems had been shown to be poor and leakage through gaps was known to dominate the protection level. Two masks from the disposable procedure/surgical mask group were evaluated, the PrimaGard Level 1 Barrier PG4-2331 with ties (five replicates), and Model 836185 Disposable Face Mask with ear loops (eight replicates). For the KN95 mask group, both masks were evaluated; MedSup Canada (7 replicates) and the TAIDAKANG (7 replicates). The N95 FFR group is represented by the 3M 9210 for total inward leakage (eight replicates), as the maximum penetration was one of the lowest (~1.6%) at the smallest particle diameter 0.043 μm. Five N95 FFRs including Halyard PFR 95, 3M 9210, North Safety 7130, Gerson 2130 and 3M 8210 (overall total of 30 replicates) were considered for the QNFT fit factor.

**Fig 9:**
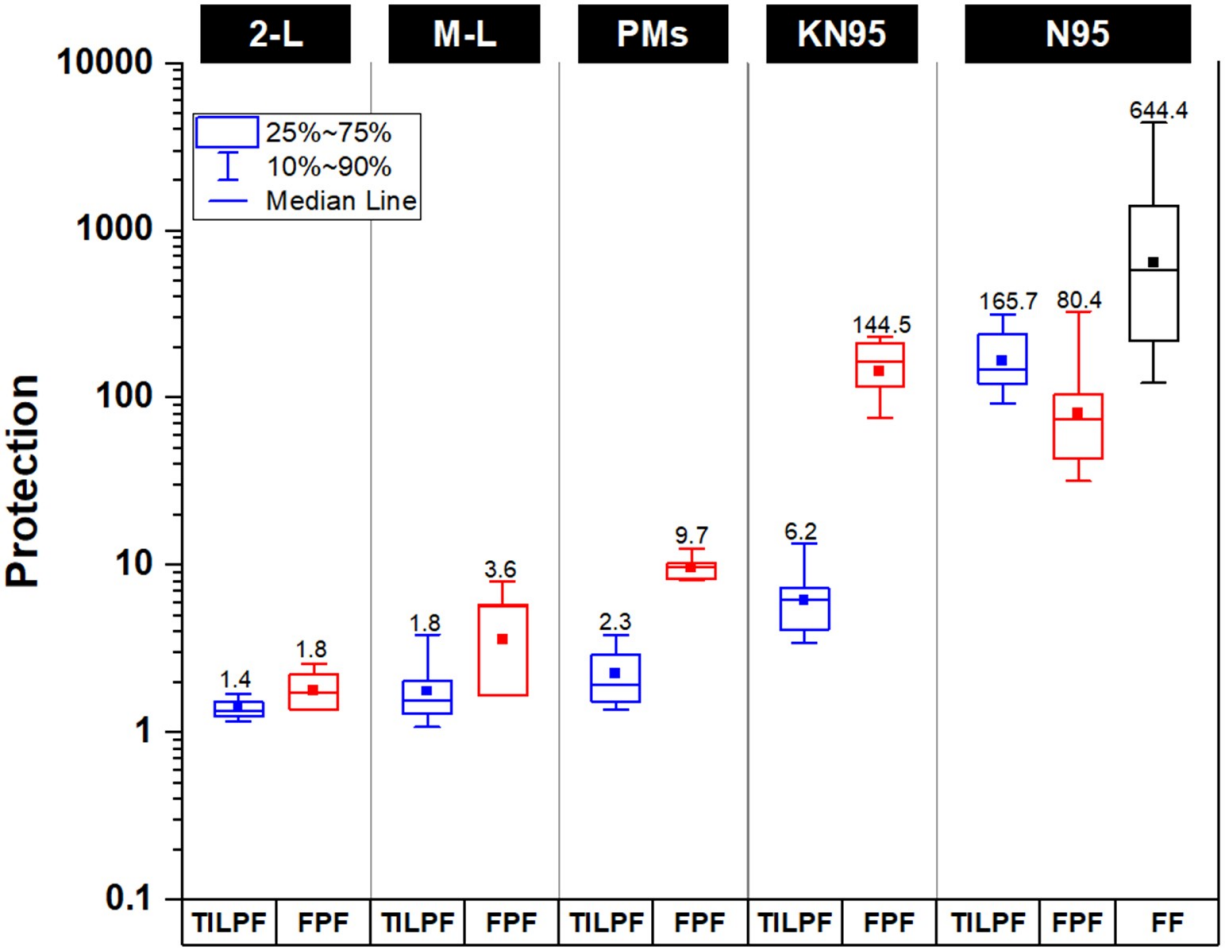
Protection performance results for the five mask groups; fabric 2-layer masks (2-L), multi-layer masks (M-L), disposable procedure masks (PMs), KN95 masks and N95 FFRs. TILPF—total inward leakage protection factor. FPF—fabric protection factor (overall protection of fabric material against entire particle size distribution). FF—fit factor (total inward leakage measured through face seal only (by QNFT). Sample sizes: n = 8 to 26 for TILPF; n = 6 to 20 for FPF; and; n = 30 for FF. The geometric mean is shown by the square marker.

Fig 9 presents a box and whisker plot of the geometric mean total inward leakage protection factor (TILPF) for each mask group and compares these values with the geometric mean FPF determined for materials that the masks in these groups may be constructed from. Also shown for comparison is the N95 FFR quantitative fit factor, which is a measure of the penetration at the face seal only. The geometric mean TILPF for the fabric 2-layer mask, fabric multi-layer mask, disposable procedure mask and KN95 mask groups was 1.42, 1.78, 2.26 and 6.20 respectively, corresponding to a total inward leakage penetration of 70%, 56.5%, 44.2% and 16.1%. Comparatively, the geometric mean TILPF for the N95 FFR mask was 165.7 (total inward leakage penetration of 0.60%). A statistical comparison was completed on the TILPF and FPF results. TILPF data is derived from a log-normal distribution therefore values were first converted to total inward leakage penetration (1/TILPF). The mean inward leakage penetration data for the five groups were assessed for normality and passed at P = 0.05 (Shapiro-Wilk normality test). A one-way analysis of variance (ANOVA) pair-wise means comparison with the Bonholm Test was performed on the different mask groups and the results are provided in S1 Appendix. Statistical analysis confirmed that the N95 FFRs provided a significantly higher TILPF compared to the KN95 masks, and in turn, the KN95 masks provided a higher level of protection compared to the other mask groups.

The geometric mean FPF for the fabric 2-layer mask, multi-layer mask, disposable procedure/surgical mask, KN95 mask and N95 FFR groups was 1.78, 3.61, 9.73, 144.5 and 69.8 respectively. The mean fabric penetration (1/FPF) data for the five mask groups were assessed for normality and the multi-layer mask group and the KN95 mask group failed at P = 0.05 (Shapiro-Wilk normality test). Accordingly, a non-parametric Kruskal-Wallis ANOVA with the Dunn’s Test was performed on the different mask groups and the results are provided in S1 Appendix. Statistical analysis confirmed that there was no significant difference in the filtration efficiencies between the N95s and KN95s mask group, and the filtration efficiencies for the KN95 and N95 respirators were significantly higher than the procedure mask group. In turn, the filtration efficiency of the procedure mask group was significantly higher than the fabric 2-layer and multi-layer group, and there was no significant difference in the filtration efficiencies between the fabric 2-layer and multi-layer group.

Comparatively, the higher TILPF for the N95 FFR group, by a factor of 27 to 117, is directly related to their design and certification, which requires that they fit and seal to the face better, more sizes are available for wearers to choose from, and the wearers are generally trained in their use. The geometric mean N95 FFR FF results (~644) shown in Fig 9, which only measure inward leakage at the face seal, further underline the exceptional performance that can be achieved with materials and face piece designs engineered for filtration efficiency and fit. The FF measured here is equivalent to a face seal efficiency of 99.84%.

The reason the N95 FFR TILPF is higher than the N95 FPF is likely due to the lower air flow through the N95 FFRs when conducting the total inward leakage test (resting breathing rate assumed to be 10 to 13 L/min [91] as compared to the nominal flow rate of 17 L/min for the aerosol swatch penetration tests. Protection levels obtained for individual fabric masks and disposable procedure masks compared to the N95 FFR are provided in S1 Table in S1 Appendix. Filter penetration (1/FPF), TILPF and total inward penetration data for each of the mask groups are provided in S2-S4 Tables in S1 Appendix, respectively.

## 4 Discussion

Our study has clearly shown that there is a variation in the level of particle penetration through materials used in the construction of masks. Furthermore, our evaluation of the TILPF on masks actually worn by trained users demonstrated that mask fit with an effective face seal is far more important to reducing total inward leakage and protecting the wearer than the filtration efficiency of the material used in the construction of the mask. Adding additional filtering layers to cloth mask concepts did not improve the total inward leakage performance, nor did single higher filtration efficiency materials in procedure masks. We also highlight the same trend for KN95 masks: although material filtration efficiencies were on par with N95 FFR materials, the TILPF were substantially lower and did not meet the required TILPF level as certified for KN95 respirators when tested against our volunteer sample size.

### 4.1 Particulate penetration through mask fabrics

Filtration efficiencies of fabrics that may be incorporated into reusable cloth masks reported in the literature are highly variable. Zangmeister *et al*. [62] found minimum filtration efficiencies were <32% (penetration >68%) for a wide range of fabrics. These authors make the point that filtration efficiency is a complex interplay between fibre type, mass and how the fabric is made (weave, melt blown, or bonded). A systematic investigation by Drewnick *et al*. [81] of 44 fabrics observed filtration efficiencies varying from ~10% to ~80% (penetration from 20% to 90%). Rengasamy’s *et al*. [96] study saw filtration efficiencies for fabrics/materials from 10–60%. Shakya *et al*. [97] noted filtration efficiencies for fabrics from 40% to 80% (penetration from 20% to 80%). Hill *et al*.’s [4] study also demonstrated that homemade face coverings provided as-worn filtration efficiencies of 15–40% (fabric protection factors of 1.18 to 1.67). A recent study by Konda *et al*. [98] looked at the aerosol filtration efficiency of common fabrics used in homemade cloth masks using a particle size distribution from 0.01 μm to 6 μm, and the authors make several points in a response to the editor [99] that increasing mask area to improve protection will lead to diminishing returns because of the tendency for more gaps to occur where the mask contacts the face, and focusing on cloth filtration properties alone may be of limited use when gaps provide alternate penetration routes. Accordingly, the broad range of filtration efficiencies highlighted in the literature agrees well with our findings for fabric 2- and multi-layer masks.

The maximum mean penetration for the materials in each mask group varied from 91.9% through the fabric 2-layer group to as low as 2.28% through the KN95 mask group (see Figs 4–8). The geometric mean FPF of common apparel materials used in 2-layer mask was 1.78 (GSdev 0.54). The geometric mean FPF for the multi-layer mask group was higher at 3.61 (GSdev 2.58), as expected with the addition of a third filtering layer, but more variable reflecting the differences in filtration efficiency of the layers considered in this study. Indeed, the range in filtration efficiency could be far greater in multi-layer masks employed by the public than we observed due to the many options that could be considered for a third filtering layer. Overall particle penetration through these two mask groups was 56.3% for the fabric 2-layer mask materials and 28% for the multi-layer mask materials.

Disposable procedure mask materials provide higher filtration efficiency, affording a geometric mean FPF of 9.73 (GSdev 1.60), corresponding to 10% penetration. Davies *et al*. [3] measured the filtration efficiency of a surgical mask at 90%-95%, similar to our results. Not surprisingly, filtering materials used in N95 FFRs and KN95 masks were superior to the filtering materials used in the other mask groups with a geometric mean FPF of 69.8 (GSdev 2.23) and 144.5 (GSdev 1.71) respectively, reducing aerosol penetration over the entire particle distribution to 1.4% and 0.7%. Thus, these mask types with high filtration efficiency materials are on average ~45 to 80 times more effective at removing particulates from an air stream than the fabric 2-layer materials and ~8 to 15 times more effective than the disposable procedure mask materials.

#### 4.1.1 Penetrated particle number and pathogen exposure

The number of viable SARS-CoV-2 to cause infection via an inhalational exposure is not presently known. In the following we discuss the potential pathogen exposure related to the particle penetration through the mask materials investigated here. The KN95 mask material is excluded because it has a filtration efficiency similar to the N95 FFR. The number of penetrated particles was converted to penetrated particle volume to facilitate an estimate of the potential number of virions in an exposure, assuming various viral concentration loadings. Simulated Gaussian distributions, representing an external respiratory aerosol challenge, were defined with the following number mean (median) particle diameters (0.3, 0.5, 1.0, 2.0 and 3.0 μm), and standard deviation of 0.7 (equivalent to a geometric standard deviation of 2.0) (S7 Fig in S1 Appendix). Each distribution contained a total of 20,000 particles. This is consistent with Asadi et al.’s [9] experimentally measured distribution for rate of particle generation speaking at a moderate volume, which for an extended size distribution comparable to ours (0.03 μm to 4.97 μm), would equate to ~19.6 particles/s. We infer that particles are dehydrated nuclei at their equilibrium state and that the concentration of virus entrained in a particle is dependent on the volume of the particle, but constrained by a maximum face centred cubic packing arrangement (74%).

Our analysis confirmed that the number of potential penetrated virion decreased as the filtration efficiency improved. For each mask group the lowest virion penetration occurred for the particle diameter 0.3 μm whilst the highest virion penetration occurred at different diameters: 3 μm for the fabric 2-layer mask, 2 μm for the multi-layer mask, 3 μm for the disposable procedure mask and 1 μm for the N95 FFR. A synopsis of the results is provided in Table 2 showing the percent reduction in penetrated virion for each mask group compared to the fabric 2-layer mask for various particle diameters at two different viral concentrations. A more comprehensive treatment of the data is provided in S1 Appendix (see also S8 Fig and S6-S9 Tables in S1 Appendix).

**Table 2:** Percent reduction in penetrated virion for each mask group compared to the fabric 2-layer mask: Results for 0.3 μm particle diameter at a viral concentration of 0.01%, and results for the particle diameter associated with the maximum virion penetration at 1.0% concentration.

Mask type		Viral concentration (0.01%)			Viral concentration (1%)		
	Mask Material FPF	Number mean particle diameter (μm)	Number penetrated virion	Percent reduction in penetrated virion compared to fabric 2-layer mask group	Number mean particle diameter (μm)	Number penetrated virion	Percent reduction in penetrated virion compared to fabric 2-layer mask group
**Fabric 2-layer**	1.8	0.3	168.3	-	3	899,630.1	-
**Multi-layer**	3.6	0.3	68.3	59	2	62,086.1	93
**Procedure**	9.7	0.3	6.72	96	3	4,415.9	99.5
**N95 FFR**	69.8	0.3	1.4	99.2	1	474.4	99.95

There is a substantial range in the estimated number of virion entrained in the particle volume that could potentially penetrate the four mask groups. The pathogen inhalation hazard that these represent is clearly dependent on, and increases, as the filtration efficiency of the material decreases, and it generally increases as the number mean particle diameter of the challenge aerosol distribution increases. The implication is that for masks like the fabric 2-layer mask, which may let high numbers of larger particles penetrate, the potential number of penetrated virion may also be high. We do not suggest that our estimate of the numbers of penetrating virion be taken as absolute. Our assumption of a direct dependence of the number of virion in a particle on the volume of a particle has not been verified, but given the broad range in particle size (sub-micron and larger) reported to occur in respiratory events, there seems to be no *a priori* reason for virus to be preferentially entrained in any size particle, and one might expect that rapid evaporation of particles following emission may lead to a concentration of virion in smaller diameter particles. This analysis clearly elucidates the importance of materials having high filtration efficiency to remove aerosol large enough to entrain discrete virion.

### 4.2 Mask total inward leakage protection factors

Aerosol present in the environment of the size range investigated here can readily pass through even small gaps of millimeter size, which may not be immediately evident to the eyes, but nevertheless represent openings on the order of one thousand times larger than the aerosol. Larger gaps are unhindered channels for aerosol to penetrate inside the face covering and potentially be inhaled.

#### 4.2.1 2-layer, multi-layer and disposable procedure mask group

Our study highlights that the average TILPF performance of 2-layer, multi-layer and disposable procedure masks worn by trained users is <2.5, with the 2-layer cloth mask having a mean of only 1.4. We found a difference of the means for the TILPF between the fabric 2-layer (1.42) and disposable procedure mask group (2.26), although not the multi-layer group (1.77), but the improvement was small at best, and it is not possible to know whether such a difference was due to the filtration efficiency or the obvious difference in design between the mask groups. Most notably, the higher FPF for the disposable procedure mask group by a factor of 2.7–5.4, had minimal impact on the TILPF measured for this group. Overall the mask protection efficiencies are <60%. The primary reason that such low TILPFs were observed is that all masks of this general design do not seal to the face, leaving gaps through which particulates may readily penetrate. At these levels of performance, individuals wearing a fabric 2-layer mask would be exposed to 70% of aerosol in the environment whilst those wearing a disposable procedure mask 44%. Lee *et al*. [59] found that the overall geometric mean PFs of three models of surgical procedure masks selected at random from a larger group of nine models, was 2.4, which was nine times lower than the protection factors determined for N95 FFRs. Similarly, a recent study by Sickbert-Bennett *et al*. [84] showed PF results where they evaluated multiple brands of surgical and procedure face masks on one test subject, obtaining mean values of 3.5 and 1.6 respectively.

#### 4.2.2 KN95 mask group

KN95 masks, which ostensibly are of higher quality given their external standard rating, including higher filtration efficiency, afforded a TILPF performance of 6.2 (overall particle penetration of 16.1%). However, this is a factor of 27 lower than the TILPF measured for the N95 FFR group. It is particularly evident that the high filtration efficiency of the KN95 mask materials (FPF = 144.5; 14.9 times higher than the disposable procedure mask group) do not appreciably augment the achievable TILPF. Thus, although our results show that the KN95 mask group provides a higher TILPF relative to the disposable procedure mask group, it may be difficult to achieve an effective face seal with KN95 masks on the same level as a N95 FFR. A recent study by Hill *et al*. [4] showed that qualitatively fitting a KN95 and N95 FFR to a headform resulted in a FFR efficiency of only ~40%, but when the KN95 FFR was sealed to the headform with adhesive, the efficiency increased to 96.7%.

Although the KN95 respirators are supposed to be certified to a total inward leakage requirement of ≤ 8% [89] (equivalent to a TILPF of ≥12.5), both KN95 respirators in this study measured ~16% penetration (i.e., a geometric mean TILPF of ~6.2). Moreover, both KN95 respirators were not able to achieve the stated inward leakage protection requirement for any of the seven volunteers tested. The lower than expected inward leakage protection may be due to differences in facial anthropometrics of the volunteers tested in another country as compared to the volunteers tested in our study, or as manufactured, they may not actually meet the requirement (as noted by the CDC for some KN95 models that do not meet filtration efficiency requirements [100]). Although the inward leakage protection was lower than expected for the KN95s, they still provided protection 3 to 4 times higher than the other mask groups (excluding the N95 respirators).

#### 4.2.3 N95 FFR group

As expected, in terms of mask performance for total inward leakage, the N95 FFR group significantly outperformed the other mask groups with a geometric mean TILPF of 165.7. This corresponds to an overall FFR (mask) particle penetration of 0.6% (mask protection efficiency of 99.4%), and may reduce an individual’s exposure to aerosol to <1% of the amount present in the environment. Substantially higher protective performance was achievable with the N95 FFR group because, in this instance, i) each wearer was quantitatively fitted according to CSA [76] to obtain a PF ≥100 prior to the total inward leakage test being conducted, and ii) the design of a N95 FFR provides for a more effective face seal. The geometric mean for the quantitative fit tests, which only measured leakage at the face seal, was a factor of 3.9 higher than the TILPF, at 644. Accordingly, a well-fitted N95 FFR worn by a competent, knowledgeable user can achieve a face seal efficiency of 99.84%, reducing inward leakage at the mask-skin interface by as much as a factor of ~27 to ~118 when compared to KN95 masks and fabric 2-layer masks respectively.

### 4.3 Mask quality factor assessment

**Fig 10:**
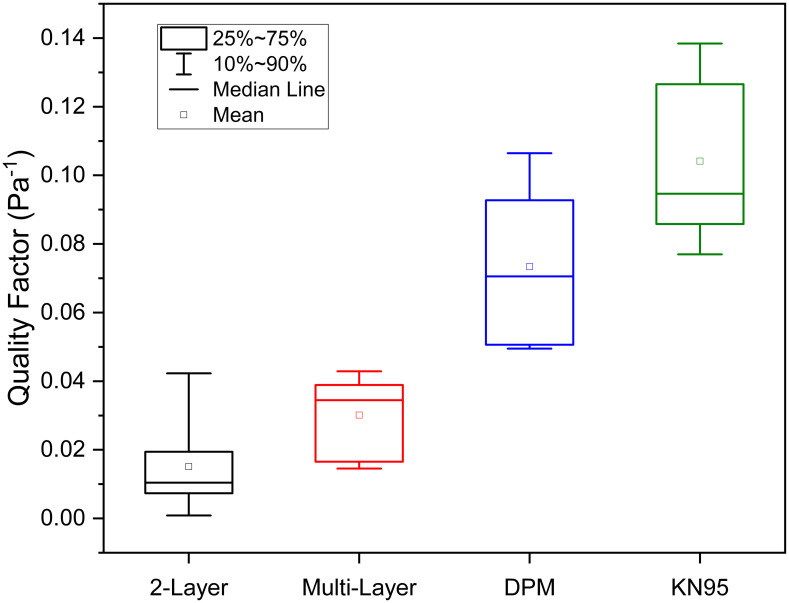
QF values for the fabric 2-layer, multi-layer, disposable procedure/surgical and KN95 mask groups. As the face velocities used in filtration testing for the N95 FFR group were dissimilar to the other three mask groups, their QF values were not plotted for comparison in this figure. DPM = disposable procedure mask.

Pressure drop may be a property that helps contribute to masking compliance, in that a mask that is easier to breathe through feels more comfortable and less burdensome. The Quality Factor (QF) is commonly used to compare the performance of materials used to filter particulates under similar experimental conditions [62–81];

$$QF=\frac{-\text{ln}\left(1-FE/100\right)}{\Delta P}$$ (3)

where *FE* is the filtration efficiency and 1-*FE* equates to 1/FPF (from our experimental measurements), and *ΔP* is the measured inhalational pressure drop (Pa). Fig 10 provides a comparative box and whisker plot of the QF values for the fabric 2-layer, multi-layer, disposable procedure/surgical mask and KN95 mask groups. The QF data for the four groups were assessed for normality and passed at P = 0.05 (Shapiro-Wilk normality test). An ANOVA pair-wise means comparison (Tukey test) determined that there was a significant difference of the mean QF between the fabric 2-layer and multi-layer mask groups and both the disposable procedure mask group and the KN95 mask group at the P = 0.05 level. There was also a significant difference of the mean QF between the disposable procedure mask group and the KN95 mask group at the P = 0.05 level. However, there was no significant difference of the means between the fabric 2-layer and multi-layer mask groups. It is evident that cloth masks, whether 2-layer or multi-layer, due to high particulate penetration, have a significantly lower Quality Factor than the disposable procedure mask group and the KN95 mask group. Although the pressure drop across the KN95 mask materials is somewhat higher than the procedure mask materials the substantially higher FPF of the KN95 in our case, results in a higher QF.

### 4.4 Preventive health measure guidance

Given that fabric and disposable procedure masks are, by simplest definition, a piece of material held in position over the nose and mouth, it may be stated reasonably confidently that an overall total inward leakage protection level of 1.5 to 2.5 is likely the upper level of performance that can be anticipated from basic designs of this nature. Limitations of effectiveness notwithstanding, it may be argued that the alternative of no one wearing a mask is considerably less desirable; some level of respiratory protection is better than none and a mask with a protection factor of 2 will reduce the level of aerosol a wearer is exposed to by 50%. Studies have shown that materials with higher filtration efficiency may reduce the outward emission of particles produced during respiratory activities through masks [63]. Accordingly, the level of respiratory protection improves further under conditions where everyone wears a mask. A review article by Kwong *et al*. [101] has summarized a wide range of studies to provide guidance for both the public to select the best materials for face masks, and for future researchers to rigorously evaluate and report on mask material testing.

We suggest that there are preferred options for mask materials other than common apparel fabrics in 2-layers. Furthermore, the range of options for incorporating a third filtering layer are numerous, and filtration efficiencies may be highly variable. Thus, multi-layer masks may provide little additional filtration above that of a 2-layer mask unless specifically tested. The multi-layer masks we investigated incorporating a polypropylene fabric layer were indistinguishable in terms of penetration from fabric 2-layer masks. Integrating a PM 2.5 filter element into a multi-layer mask may provide a filtration efficiency on par to materials used in N95 FFRs. Although not included for brevity, we measured FPFs for multi-layer masks with a PM 2.5 filter insert of ~71. However, the TILPF for masks with these inserts was only 1.8, similar in performance to the fabric 2-layer masks, thus they afforded no additional protection at the system level (Fig 9). Moreover, fabric masks with a PM 2.5 filter insert were found to have an inhalation pressure drop a factor of three higher than disposable procedure masks (see S1 Table in S1 Appendix), resulting in a markedly lower QF of 0.04, and more in line with the other multi-layer masks, despite their significantly higher filtration efficiency. In addition, a higher pressure drop may also be partially responsible for mask leakage; at least during exhalation. Materials used in commercially available disposable procedure masks generally have a higher filtration efficiency than the fabric 2-layer and multi-layer mask groups. We highlight that the disposable procedure/surgical mask group offers higher filtration efficiency and lower pressure drop, resulting in a higher QF (see Fig 10). Although this type of mask offers little additional total inward leakage protection over cloth masks, improving the filtration efficiency against particles >0.1 μm in exhaled respiratory air will reduce the penetrating particle volume and potential viral concentration therein from concentrating in the environment where people gather. Accordingly, these masks are a good choice for widespread masking guidance due to their improved performance, commercial availability and low cost.

The use of “N95-like” masks approved under standards from other countries [89], such as the KN95 protective masks evaluated here, are also an option for masking use in the general public. We found that the KN95 mask performance of the group that we investigated was superior to the disposable procedure mask group both in terms of TILPF (factor of 2.7 higher) and particularly the FPF (factor of 14.9 higher), with the only shortcoming being the low TILPF compared to the N95 FFR group. The mean mask Quality Factor was statistically higher than the disposable procedure mask group.

It is nonetheless, important to point out that the performance of other models of KN95 masks has been found to be inconsistent, making it difficult to know whether a given product actually meets the specifications of the standard to which it was supposedly manufactured. The two KN95 masks that we investigated in this study met the Chinese standard GB2626-2006 for filtration performance. However, our research group consists of knowledgeable, trained users and evaluators of masks/respirators with access to state of the art measurement instrumentation and peer reviewed studies. In a summary of filtration results made available by CDC on 59 models of KN95 masks against the KN95 filtration standard (China GB2626-2006) [100], they found that 37% provided a filtration efficiency above 95%, 29% had a filtration efficiency below 95% and 34% of the models had some masks within each assessment that were above and some below 95%. The study also showed filtration variability within each model varied from as low as 0.09% to as high as 77.50%. Users who do not understand the limitations of face masks should refer to impartial organizations such as the CDC for advice on purchasing respirators from other countries, where information on the filtration performance of non-NIOSH approved respirator models is provided [102], along with advice to the consumers on purchasing respirators from other countries such as the KN95s [103].

As one would expect, N95 FFRs, given their certification requirements, were shown to have particle penetration <2% and the highest level of inward leakage protection, the latter far exceeding that of the cloth, procedure and KN95 mask groups. Accordingly, they remain the best option to protect individuals from exposure to aerosol in high risk settings, and the environment from individuals possibly shedding infectious virus in their respiratory aerosol. Kahler and Hain [104] suggest that it is very important to differentiate between mouth-and-nose covers, surgical masks and particle filtering respirators, because they vary substantially in their fundamental protection properties. They stress that to achieve effective self-protection in a virus-contaminated environment, masks with particle filtering properties (FFP2/KN95/N95) are absolutely necessary.

## 5 Conclusion

In this study we have reported on the fabric filtration efficiency and total inward leakage protection factor for five groups of masks of relevance to the SARS-CoV-2 pandemic: reusable, fabric two- and three-layer masks, disposable procedure/surgical masks, KN95 masks and N95 FFRs. Our study has clearly shown that there is a variation in the level of particle penetration through materials used in the construction of masks. This is a function of the physical characteristics of the filtering layer, including porosity, fibre type, fabric/membrane structure, as well as electrostatic properties. The variation may be substantial within masks of the same type and even more pronounced when comparing across masks of different technologies. Cloth materials in general have the highest particle penetration. Adding two or more layers may reduce the amount of penetration but the different material options for a third filtering layer may actually increase the possible variation in expected performance. Thus, multi-layer masks may provide little additional filtration above that of a 2-layer mask unless specifically tested. Materials used in disposable procedure masks afford a higher level of protection against particle penetration. KN95 and N95 FFR materials provide the highest degree of filtering performance, in line with their specification standards. However it is important to point out that the filtration performance of other models of KN95 masks has been found to be inconsistent and not meet the specifications of the standard to which it was supposedly manufactured. Thus, it is recommended that consumers obtain information from the CDC on the filtration performance of a specific model of KN95 mask before purchasing.

Of particular note from this study is the evidence demonstrating that mask fit with an effective face seal is far more important to reducing total inward leakage and protecting the wearer than the filtration efficiency of the material used in the construction of the mask. Adding additional filtering layers to cloth mask concepts did not improve the total inward leakage performance. Moreover, despite the considerably higher filtration efficiency of the materials employed in the disposable procedure mask group, the total inward leakage protection factor for these types was only marginally improved. Although KN95 masks provided TIL protection ~3 times greater than the disposable procedure masks, it was still ~27 times lower than the TIL protection provided by N95 FFRs. Face seal leakage plays a dominant role in how effective a mask is protecting a wearer from inhalation of aerosol and N95 FFRs were the only mask group investigated that provided not only high filtration efficiency but high total inward leakage protection, and remain the best option to protect individuals from exposure to aerosol in high risk settings.

The Mask Quality Factor and total inward leakage performance are very useful to determine the best options for masking. However, it is highly recommended that testing is undertaken on prospective products, or guidance is sought from impartial authorities, to confirm they meet any implied standards.

## Supporting information

S1 Appendix (DOCX)
