# Supplementary material for: The protective performance of reusable cloth face masks, disposable procedure masks, KN95 masks and N95 respirators: Filtration and total inward leakage
Source: PLoS One. 2021 Oct 6;16(10):e0258191. doi: 10.1371/journal.pone.0258191 (PMC8494377; doi:10.1371/journal.pone.0258191)
Supplement: S1 Appendix — (DOCX) [file pone.0258191.s001.docx]

# S1 Appendix

**S1 Table. Fabric protection factor, inhalation pressure drop and face velocity data measured for the various fabrics systems that were evaluated in this study. The Quality Factor is also calculated for each sample.**

| **Fabric System** | **Fabric Protection Factor (FPF)** | **Face Velocity (cm/s)** | **Inhalation Pressure Drop (Pa)** | **Quality Factor (Pa^-1^)** |
| --- | --- | --- | --- | --- |
| ***Polyester fabric (2 layers)** | **1.37** | 4.87 | 24.5 | 0.0128 |
| ***Quilt batting (2 layers)** | **2.60** | 4.87 | 22.6 | 0.0423 |
| ***Nylon fabric (2 layers)** | **1.39** | 4.87 | 375.7 | 0.0009 |
| **Cotton fabric variant 1 (two layers)**  **(CC)** | **1.37** | 4.87 | 39.2 | 0.0080 |
| **Silk fabric (2 layers)**  **(SS)** | **2.24** | 4.87 | 109.8 | 0.0073 |
| **Quilt batting/cotton (2 layers) (QC)** | **2.10** | 4.87 | 38.2 | 0.0194 |
| **Cotton/Furnace Filter/Cotton**  **(3 layers) (CFFC)** | **8.03** | 4.87 | 49.1 | 0.0424 |
| **Cotton/2x quilt batting/cotton (4 layers) (C2QC)** | **5.82** | 4.87 | 48.0 | 0.0367 |
| **Cotton/PM 2.5 filter/rayon (CFIR)** | **111.10** | 4.87 | 109.8 | 0.0429 |
| **Cotton/Disposable Procedure Mask (DPM)/Cotton liner**  **(CDPMC)** | **5.72** | 4.87 | 60.8 | 0.0287 |
| **Bi-laminate in-house technical research material (ITRM)** | **96.6** | 4.87 | 260.8 | 0.0175 |
| **Cotton/PM2.5/Cotton Liner** | **45.36** | 4.87 | 98.1 | 0.0389 |
| **Quilt cotton/electret filter membrane/Cotton Knit**  **(QC_A#2_Cotton Knit)** | **5.71** | 4.87 | 50.5 | 0.0345 |
| **Quilt Cotton/Brown Polypropylene ('craft')/Cotton Knit**  **(QC_Brown-PP_CK)** | **1.67** | 4.87 | 35.3 | 0.0145 |
| **Quilt Cotton/Green Polypropylene ('craft')/Cotton Knit**  **(QC_Green-PP_CK)** | **1.68** | 4.87 | 31.4 | 0.0165 |
| **Quilt Cotton/Shopping bag Polypropylene/Cotton Knit**  **(QC_Shopping Bag-PP_CK)** | **1.85** | 4.87 | 38.3 | 0.0161 |
| **Disposable procedure mask**  **(Henan Liwei)** | **12.57** | 4.87 | 50.0 | 0.0506 |
| **Disposable procedure mask**  **(PG4-1200)** | **8.20** | 4.87 | 39.2 | 0.0537 |
| **Disposable procedure mask**  **(PG4-1273)** | **9.70** | 4.87 | 24.5 | 0.0927 |
| **Disposable procedure mask (Vanche)** | **8.27** | 4.87 | 42.7 | 0.0495 |
| **Disposable procedure mask (PG4-2001)** | **10.32** | 4.87 | 26.7 | 0.0874 |
| **Disposable procedure mask**  **(PG4-2331)** | **9.97** | 4.87 | 21.6 | 0.1065 |
| **N95 North Safety 7130** | **166.70** | 2.85 | 52.0 | 0.0984 |
| **N95 Halyard FLUIDSHIELD 2** | **92.80** | 1.10 | 45.5 | 0.0997 |
| **Gerson 2130** | **41.53** | 1.44 | 51.2 | 0.0728 |
| **N95 3M 8110s** | **48.70** | 6.69 | 51.2 | 0.0759 |
| **N95 3M 1870** | **86.60** | 1.73 | 56.4 | 0.0791 |
| **N95 3M 9210** | **243** | 1.73 | 54.9 | 0.100 |

**S1 Fig. An example of the variability of the aerosol swatch penetration data for repeat measurements (up to four upstream and downstream measurements), adjusting for small changes in the challenge concentration.**

**S2 Fig. Values for number penetration as a function of aerodynamic particle diameter, for each mask tested in the fabric 2-layer mask group.**

**S3 Fig. Values for number penetration as a function of aerodynamic particle diameter, for each mask tested in the multi-layer mask group. Two multi-layer masks incorporating a PM 2.5 filter element (dashed lines) are provided for reference, but were not included in the mean penetration profile of the 3-layer masks due to their significantly higher filtration efficiency.**

**S4 Fig. Values for number penetration as a function of aerodynamic particle diameter, for each mask tested in the disposable procedure/surgical mask group.**


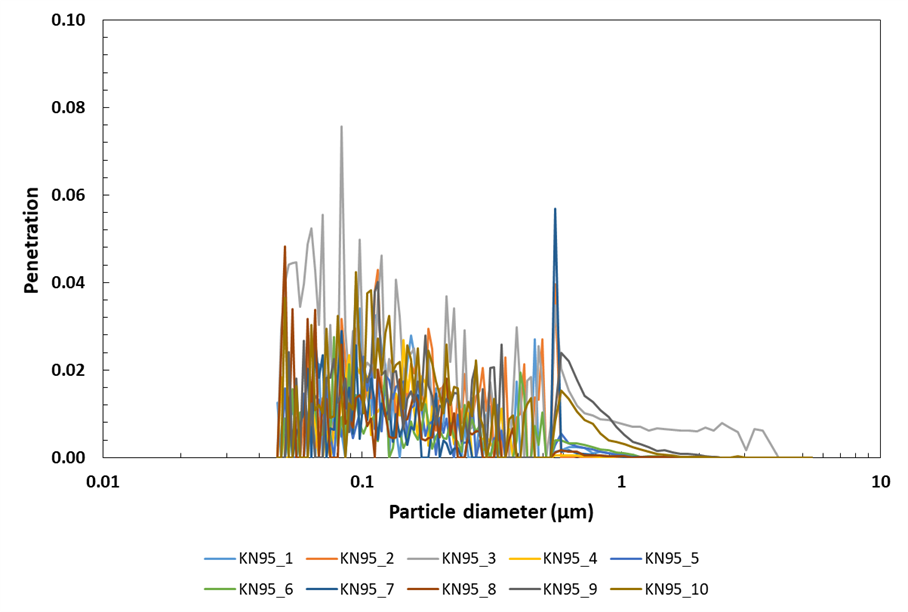


**S5 Fig. Values for number penetration as a function of aerodynamic particle diameter, for each mask tested in the KN95 group. Note, y-axis scale maximum of 0.1.**

**S6 Fig. Values for number penetration as a function of aerodynamic particle diameter, for each mask tested in the N95 FFR group. Note, y-axis scale maximum of 0.2.**

# Details of Masks Evaluated

**S2 Table. List of the make/model and manufacturer and/or source of all the masks and fabric material tested in the study. The masks/fabric material were either purchased commercially, provided by different agencies or were homemade. The type of test (filtration efficiency (FE), total inward leakage protection factor (TILPF) testing and/or Quantitative fit testing (QNFT)), performed on each type of mask/fabric material is listed below.**

| **Make / Model** | **Distributor / Manufacturer / Source** | **Testing** |
| --- | --- | --- |
| **2-layer mask group** | | |
| Polyester fabric | Homemade | FE |
| Quilt batting | Homemade | FE |
| Nylon fabric | Homemade | FE |
| Cotton fabric variant 1 (CC) | Department of National Defence, Canada | FE and TILPF |
| Cotton fabric variant 2 | Department of National Defence, Canada | TILPF |
| Silk fabric (SS) | Homemade | FE and TILPF |
| Quilt batting/Cotton (QC) | Homemade | TILPF |
| **Multi-layer mask group** | | |
| Quilt cotton/electret filter membrane /Cotton Knit (QC_A#2_Cotton knit) | Public Health Agency of Canada^a^ | FE |
| Cotton/Furnace Filter/Cotton  (3 layers) (CFFC) | Homemade^b^ | FE and TILPF |
| Cotton/2x quilt batting/cotton (4 layers) (C2QC) | Homemade | FE and TILPF |
| Cotton/PM 2.5 filter/rayon (3 layer) (CFIR) | Weddingstar (Medicine Hat, Canada) | FE and TILPF |
| Cotton/Disposable Procedure Mask (DPM)/Cotton Liner (3 layer) (CDPMC) | Homemade | FE |
| Cotton/PM2.5/Cotton Liner | Homemade | FE and TILPF |
| Quilt Cotton/Brown Polypropylene ('craft') /Cotton Knit (QC_Brown-PP_CK) | Public Health Agency of Canada^c^ | FE |
| Quilt Cotton/Green Polypropylene ('craft')/Cotton Knit (QC_Green-PP_CK) | Public Health Agency of Canada^c^ | FE |
| Quilt Cotton/Shopping bag Polypropylene/Cotton Knit (QC_Shopping Bag-PP_CK) | Public Health Agency of Canada | FE |
| Bi-laminate in-house technical research material (ITRM) | Department of National Defence, Canada | FE |
| **Procedure/surgical mask group** | | |
| Model PG4-1200 PrimaGard (PG4-1200) | Medical Products Inc. (Edmonton, Canada) | FE |
| Model PG4-1273 PrimaGard Level 3 Barrier (PG4-1273) | Medical Products Inc. (Edmonton, Canada) | FE |
| Model PG4-2001 PrimaGard Surgical Mask Level 1 Barrier (PG4-2001) | Medical Products Inc. (Edmonton, Canada) | FE |
| Model PG4-2331 PrimaGard Level 1 Barrier (PG4-2331) | Medical Products Inc. (Edmonton, Canada) | FE and TILPF |
| Model 836185 Disposable Face Mask (Henan Liwei) | Henan Liwei Biological Pharmaceutical Co. Ltd | FE and TILPF |
| Vanch Disposable Medical Face Mask (Vanche) | 2020 Beifa Group Co. Ltd., Ningbo China | FE |
| **KN95 mask group** | | |
| MedSup Canada KN95 Protective Face Mask | MedSup Canada (distributer) (manufactured in China) | FE and TILPF |
| TAIDAKANG KN95 Protective mask | TAIDAKANG (manufactured in China) | FE and TILPF |
| **N95 FFR group** | | |
| Model FLUIDSHIELD 2 N95 | Halyard Health | FE/QNFT |
| Model 7130N95 | North Safety Products | FE/QNFT |
| Model 2130 N95 | Louis M. Gerson Co. | FE/QNFT |
| Model 9210 N95 | 3M™ | TILPF/QNFT |
| Model 1870 | 3M™ | FE |
| Model 8110s | 3M™ | FE |
| Model 8210 | 3M™ | QNFT |
| ^a^: “N95-like” electret filter membrane material purchased from Amazon.ca  ^b^: Furnace filter manufactured by 3M™ Filtrete furnace filter, Merv 13  ^c^: Polypropylene ‘craft’ material manufactured by smart-fab® | | |

**S3 Table. Filter penetration data (1/FPF) for each of the five mask groups.**

| **Fabric 2-layer** | **Multi-layer** | **Procedure Masks** | **KN95** | **N95 FFR** |
| --- | --- | --- | --- | --- |
| **Filter Penetration** | **Filter Penetration** | **Filter Penetration** | **Filter Penetration** | **Filter Penetration** |
| 0.385 | 0.172 | 0.103 | 0.007 | 0.024 |
| 0.730 | 0.125 | 0.100 | 0.008 | 0.028 |
| 0.719 | 0.175 | 0.097 | 0.025 | 0.021 |
| 0.730 | 0.175 | 0.122 | 0.005 | 0.013 |
| 0.446 | 0.598 | 0.080 | 0.004 | 0.011 |
| 0.483 | 0.596 | 0.121 | 0.005 | 0.009 |
| **Geomean 0.563** | 0.540 | **Geomean 0.103** | 0.004 | 0.01 |
|  | **Geomean 0.278** |  | 0.005 | 0.015 |
|  |  |  | 0.009 | 0.003 |
|  |  |  | 0.009 | 0.014 |
|  |  |  | **Geomean 0.007** | 0.031 |
|  |  |  |  | 0.023 |
|  |  |  |  | 0.011 |
|  |  |  |  | 0.012 |
|  |  |  |  | 0.04 |
|  |  |  |  | 0.017 |
|  |  |  |  | 0.048 |
|  |  |  |  | 0.002 |
|  |  |  |  | 0.031 |
|  |  |  |  | 0.005 |
|  |  |  |  | **Geomean 0.014** |

**S4 Table. Total inward leakage protection factors (TILPF) data for each of the five mask groups.**

| **Fabric 2-layer** | **Multi-layer** | **Procedure Masks** | **KN95** | **N95 FFR** |
| --- | --- | --- | --- | --- |
| **TILPF** | **TILPF** | **TILPF** | **TILPF** | **TILPF** |
| 1.52 | 1.37 | 1.92 | 7.06 | 228.79 |
| 1.43 | 1.54 | 1.3 | 13.48 | 138.13 |
| 1.13 | 1.24 | 2.24 | 3.38 | 256.25 |
| 1.18 | 1.3 | 1.53 | 6.28 | 124.66 |
| 1.7 | 1.59 | 1.39 | 6.14 | 159.79 |
| 1.63 | 4.51 | 2.07 | 3.53 | 318.97 |
| 2.82 | 4.91 | 1.81 | 14.71 | 92.27 |
| 1.69 | 3.2 | 1.42 | 6.30 | 119.92 |
| 1.25 | 1.72 | 2.92 | 5.44 | **Geomean 165.74** |
| 1.9 | 3.19 | 1.92 | 4.16 |  |
| 1.3 | 1.29 | 8.28 | 7.27 |  |
| 1.37 | 1.08 | 3.49 | 9.54 |  |
| 1.25 | 1.09 | 3.86 | 3.44 |  |
| 1.44 | 1.07 | **Geomean 2.26** | 5.65 |  |
| 1.21 | 1.42 |  | **Geomean 6.20** |  |
| 1.32 | 1.78 |  |  |  |
| 1.28 | 2.15 |  |  |  |
| 1.3 | 1.48 |  |  |  |
| 1.67 | 1.92 |  |  |  |
| 1.41 | 1.72 |  |  |  |
| 1.11 | **Geomean 1.77** |  |  |  |
| 1.25 |  |  |  |  |
| 1.32 |  |  |  |  |
| 1.25 |  |  |  |  |
| 1.53 |  |  |  |  |
| 1.49 |  |  |  |  |
| **Geomean 1.42** |  |  |  |  |

**S5 Table. Total inward leakage penetration data for each of the five mask groups.**

| **Fabric 2-layer** | **Multi-layer** | **Procedure Masks** | **KN95** | **N95 FFR** |  |
| --- | --- | --- | --- | --- | --- |
| **Total inward leakage penetration** | **Total inward leakage penetration** | **Total inward leakage penetration** | **Total inward leakage penetration** | **Total inward leakage penetration** |  |
| 0.6693 | 0.7304 | 0.5216 | 0.1416 | 0.0044 |  |
| 0.7002 | 0.6493 | 0.7694 | 0.0742 | 0.0072 |  |
| 0.8881 | 0.8032 | 0.4461 | 0.2961 | 0.0039 |  |
| 0.8503 | 0.7699 | 0.6551 | 0.1593 | 0.0080 |  |
| 0.5875 | 0.6307 | 0.7219 | 0.1629 | 0.0063 |  |
| 0.615 | 0.2215 | 0.4831 | 0.2834 | 0.0031 |  |
| 0.3547 | 0.2035 | 0.5529 | 0.0680 | 0.0108 |  |
| 0.5934 | 0.312 | 0.7047 | 0.1587 | 0.0083 |  |
| 0.7989 | 0.581 | 0.3422 | 0.1838 | **Geomean 0.0060** |  |
| 0.5251 | 0.3132 | 0.5205 | 0.2405 |  | |
| 0.769 | 0.7767 | 0.1207 | 0.1376 |  | |
| 0.7324 | 0.9255 | 0.2865 | 0.1048 |  | |
| 0.802 | 0.9214 | 0.2593 | 0.2907 |  | |
| 0.695 | 0.9374 | **Geomean 0.4432** | 0.1770 |  | |
| 0.8272 | 0.7031 |  | **Geomean 0.1614** |  | |
| 0.7581 | 0.563 |  |  |  | |
| 0.7798 | 0.4645 |  |  |  | |
| 0.7684 | 0.6759 |  |  |  | |
| 0.5999 | 0.5201 |  |  |  | |
| 0.708 | 0.5813 |  |  |  | |
| 0.9018 | **Geomean 0.5651** |  |  |  | |
| 0.7972 |  |  |  |  | |
| 0.7575 |  |  |  |  | |
| 0.7989 |  |  |  |  | |
| 0.6526 |  |  |  |  | |
| 0.6723 |  |  |  |  | |
| **Geomean 0.7034** |  |  |  |  | |

**Penetrated particle number and particle volume**

In the following we discuss the potential pathogen exposure related to the filtration efficiency of the 2-layer, multi-layer, disposable procedure and N95 FFR mask materials. The KN95 mask material is excluded because it has a filtration efficiency similar to the N95 FFR.

We defined five Gaussian log-normal aerosol distributions with the following number mean (median) particle diameters (0.3, 0.5, 1.0, 2.0 and 3.0 µm), and standard deviation of 0.7 (equivalent to a geometric standard deviation of 2.0). Each distribution contained a total of 20,000 particles. This is consistent with Asadi et al.’s [S1] experimentally measured distribution for rate of particle generation speaking at a moderate volume, which for an extended size distribution comparable to ours (0.03 µm to 4.97 µm), would equate to ~19.57 particles/s. Accordingly, two persons speaking and generating respiratory particles at this rate for 10 min (600 s) would produce a total of 23,733 particles. The simulated Gaussian distributions, representing an external respiratory aerosol challenge, are illustrated in S7 Fig. Using the experimental penetration profiles measured for each mask group, we determined the number of penetrated particles of a given diameter for each challenge distribution according to

$n_{p_{i}}=P_{E_{i}}N_{i}$ Equation (S1)

where $n_{p_{i}}$ is the number of particles calculated to penetrate through the filtering material based on the measured penetration factor $P_{E_{i}}$ for each particle diameter (bin) *i*, and *N_i_* is the corresponding number of external challenge particles. The number of penetrated particles was converted to penetrated particle volume to facilitate an estimate of the potential number of virions in an exposure assuming various viral concentration loadings in the challenge aerosol.

We consider that particles expelled during normal respiratory activities by individuals symptomatic with SARS-CoV-2 may contain viable virus at a certain concentration level. Equilibrium particle sizes are reached nearly instantaneously on emission. [S2] Hydrated respiratory particles will typically dry and shrink, the final size depending on surface curvature, solute matter and the humidity in the environment. [S3,S4] Accordingly, in the following discussion particles are considered to be dehydrated nuclei at their equilibrium state. We infer that the concentration of virus entrained in a particle is dependent on the volume of the particle, but constrained by a maximum face centred cubic packing arrangement (74%). Lee [S5] considered a simple volumetric relationship between the volume of the pathogen and the volume of the particle, but proposed minimum particle diameters based on concentration, smaller than which, would not contain virus. This seems somewhat arbitrary. Whilst for example, one virion 0.09 µm diameter is only 0.01% of the volume of a particle 1.9 µm in diameter, [S5] there is no physical reason precluding a virion from being entrained in a particle less than this diameter. Some studies suggest that the encapsulation of virus in particles may have limited dependence on the particle volume, instead their inclusion following a Poisson distribution, such that the probability of smaller diameter particles containing virus is much lower than larger particles. [S6] Presently, there is no confirming evidence that this may be the case for SARS-CoV-2 and our assessment is a more conservative approach. For a SARS-CoV-2 virion ~0.1 µm in size, [S7] a 1 µm diameter particle, for example, could contain a maximum 740 virion (assuming face centred cubic packing), a particle twice this diameter 5,920 virion whilst a particle only 0.3 µm diameter would contain just 20 virion. The lower limit in this case is a particle 0.1 µm diameter, comprised solely of one virion. Here we assume that the viral load concentration applies to the particle distribution from 0.1 µm to 5 µm. It is evident that there is the possibility for vastly larger numbers of virion to be present in penetrated distributions containing larger diameter particles. To calculate the range of potential virion exposure through each mask group with each challenge particle distribution described above, we systematically varied the viral load from 0.01% to 1%, which, from the following relationship,

$C_{v}=\frac{6}{\pi d_{p}^{3}}u_{m}\frac{V_{l}}{100}$ Equation (S2)

corresponds to 0.1413 virion/µm^3^ to 14.13 virion/µm^3^, where *µ_m_* is the maximum number of virion (face centred cubic packed) in a particle of diameter *d_p_*, and *V_l_* is the viral load in percent. These values reflect the upper end of virion concentrations that potentially could be emitted. [S8] Although to our knowledge, there is no published evidence linking clinical laboratory measurements of SARS-CoV-2 RNA (copies/ml), measured in sputum and swab samples of individuals, with the SARS-CoV-2 viral load in aerosol produced via normal respiratory activities by an infected person.

As our assessment of the exposure hazard is scalable, if we consider a much reduced viral load of 10-5 % (0.0001413 virion/µm3), for example, our mask penetration profiles further highlight that an insignificant number of virion penetrate through the N95 FFR for all simulated challenge distributions, and <1 virion penetrates through the multi-layer and disposable procedure mask groups. However, for the fabric 2-layer mask group, which has the poorest filtration efficiency, we determine 2 to 9 virion penetrate for the challenge distributions with a mean number particle diameter between 1 µm and 3 µm respectively, but <1 virion for the challenge distributions with a mean number particle diameter <0.5 µm. Our results for an exposure aerosol with a number mean penetration of 1 µm (calibrated against Asadi’s et al. [S1] particle generation data for speaking), can be scaled based on the duration of talking and/or number of people talking.


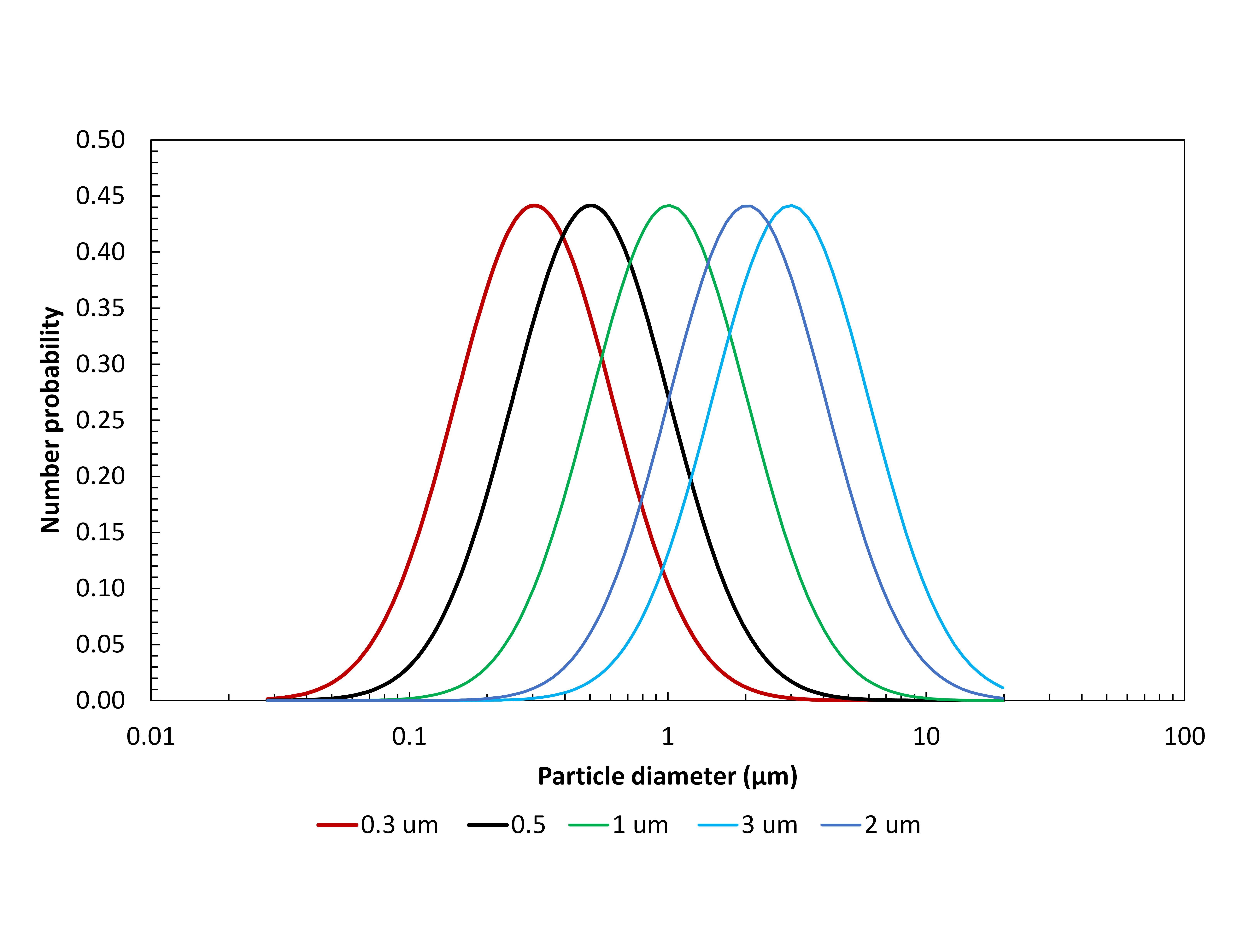


**S7 Fig. Simulated challenge aerosol distributions: log-normal Gaussian with number mean particle diameters of 0.3, 0.5, 1.0, 2.0 and 3.0 µm, and standard deviation of 0.7 (equivalent to geometric standard deviation of 2.0).**

(a) (b)


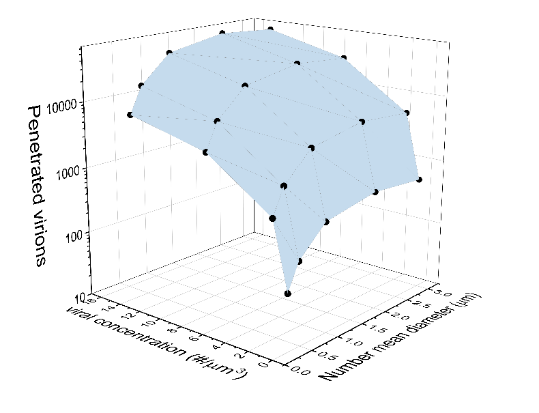

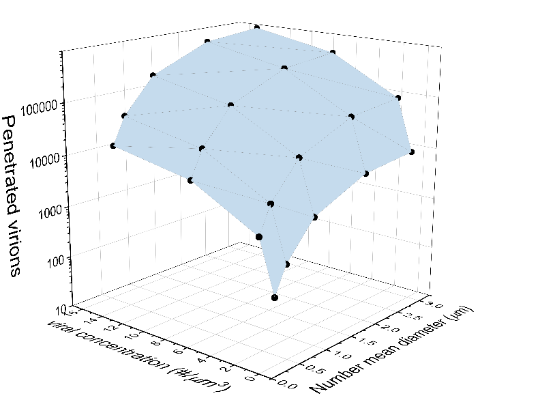

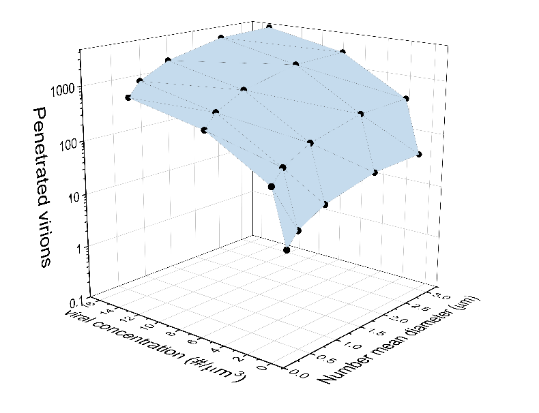

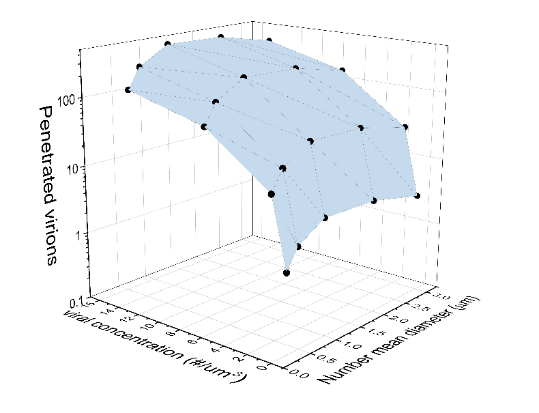


(c) (d)

**S8 Fig. Three-Dimensional surface plot showing the relationship between the number of virion penetrated (z-axis) through the four mask group penetration profiles as a function of the viral load concentration (x-axis) and number mean particle diameter (y-axis) in each aerosol challenge distribution: (a) Fabric 2-layer mask, (b) Multi-layer mask group, (c) disposable procedure mask group, and (d) N95 FFR group. Note change in scale of y-axis within the figure.**

**S6 Table. The number and volume of aerosol, and the calculated virion penetrated, through the four mask group penetration profiles (see Figure 1 to 4) as a function of the number mean particle diameter for each challenge distribution, and a viral load concentration of 0.14 virion/um^3^.**

| **Mask type** |  | **Number mean diameter (µm) of challenge distribution** | | | | |
| --- | --- | --- | --- | --- | --- | --- |
|  |  | **0.3** | **0.5** | **1** | **2** | **3** |
| Fabric 2-layer | Number aerosol penetrated | 13999.3 | 14773.9 | 14603.7 | 11393.5 | 8292.1 |
|  | % Number fraction | 95.97 | 99.20 | 99.94 | 100 | 100 |
|  | Volume aerosol penetrated (µm^3^) | 1190.9 | 3858.3 | 17270.8 | 49956.7 | 63654.6 |
|  | % Volume fraction | 99.99 | 99.99 | 100 | 100 | 100 |
|  | Virion penetrated | 168 | 545 | 2,441 | 7060 | 8996 |
| Fabric 3-layer | Number aerosol penetrated | 7937.4 | 7787.9 | 6546.2 | 3717.7 | 2056.9 |
|  | % Number fraction | 95.00 | 98.94 | 99.93 | 100 | 100 |
|  | Volume aerosol penetrated (µm^3^) | 483.53 | 1199.03 | 3059.67 | 4392.99 | 3724.54 |
|  | % Volume fraction | 99.98 | 99.99 | 100 | 100 | 100 |
|  | Virion penetrated | 68 | 169 | 432 | 621 | 526 |
| Procedure | Number aerosol penetrated | 1720.7 | 1282.7 | 755.6 | 278.3 | 122.1 |
|  | % Number fraction | 86.31 | 96.25 | 99.64 | 99.98 | 100 |
|  | Volume aerosol penetrated (µm^3^) | 47.60 | 85.30 | 160.02 | 282.72 | 312.46 |
|  | % Volume fraction | 99.87 | 99.98 | 99.99 | 100 | 100 |
|  | Virion penetrated | 7 | 12 | 23 | 40 | 44 |
| N95 | Number aerosol penetrated | 311.61 | 240.16 | 147.67 | 60.91 | 27.81 |
|  | % Number fraction | 89.91 | 97.26 | 99.74 | 99.99 | 100 |
|  | Volume aerosol penetrated (µm^3^) | 9.64 | 18.90 | 33.56 | 31.42 | 20.27 |
|  | % Volume fraction | 99.91 | 99.99 | 100 | 100 | 100 |
|  | Virion penetrated | ~1 | 3 | 5 | 4 | 3 |

**S7 Table. The number and volume of aerosol, and the calculated virion penetrated, through the four mask group penetration profiles (see Figure 1 to 4) as a function of the number mean particle diameter for each challenge distribution, and a viral load concentration of 1.41 virion/um^3^.**

| **Mask type** |  | **Number mean diameter (µm) of challenge distribution** | | | | |
| --- | --- | --- | --- | --- | --- | --- |
|  |  | **0.3** | **0.5** | **1** | **2** | **3** |
| Fabric 2-layer | Number aerosol penetrated | 13999.3 | 14773.9 | 14603.7 | 11393.5 | 8292.1 |
|  | % Number fraction | 95.97 | 99.20 | 99.94 | 100 | 100 |
|  | Volume aerosol penetrated (µm^3^) | 1190.87 | 3858.38 | 17270.80 | 49956.65 | 63654.57 |
|  | % Volume fraction | 99.99 | 99.99 | 100 | 100 | 100 |
|  | Virion penetrated | 1683 | 5453 | 24409 | 70604 | 89963 |
| Fabric 3-layer | Number aerosol penetrated | 7937.4 | 7787.9 | 6546.2 | 3717.7 | 2056.9 |
|  | % Number fraction | 95.00 | 98.94 | 99.93 | 100 | 100 |
|  | Volume aerosol penetrated (µm^3^) | 483.53 | 1199.03 | 3059.67 | 4392.99 | 3724.54 |
|  | % Volume fraction | 99.98 | 99.99 | 100 | 100 | 100 |
|  | Virion penetrated | 683 | 1695 | 4324 | 6209 | 5264 |
| Procedure | Number aerosol penetrated | 1720.7 | 1282.7 | 755.6 | 278.3 | 122.1 |
|  | % Number fraction | 86.31 | 96.25 | 99.64 | 99.98 | 100 |
|  | Volume aerosol penetrated (µm^3^) | 47.60 | 85.30 | 160.02 | 282.72 | 312.46 |
|  | % Volume fraction | 99.87 | 99.98 | 99.99 | 100 | 100 |
|  | Virion penetrated | 67 | 121 | 226 | 400 | 442 |
| N95 | Number aerosol penetrated | 311.6 | 240.2 | 147.7 | 60.9 | 27.8 |
|  | % Number fraction | 89.91 | 97.26 | 99.74 | 99.99 | 100 |
|  | Volume aerosol penetrated (µm^3^) | 9.64 | 18.90 | 33.56 | 31.42 | 20.27 |
|  | % Volume fraction | 99.91 | 99.99 | 100 | 100 | 100 |
|  | Virion penetrated | 14 | 27 | 47 | 44 | 29 |

**S8 Table. The number and volume of aerosol, and the calculated virion penetrated, through the four mask group penetration profiles (see Figure 1 to 4) as a function of the number mean particle diameter for each challenge distribution, and a viral load concentration of 7.07 virion/um^3^.**

| **Mask type** |  | **Number mean diameter (µm) of challenge distribution** | | | | |
| --- | --- | --- | --- | --- | --- | --- |
|  |  | **0.3** | **0.5** | **1** | **2** | **3** |
| Fabric 2-layer | Number aerosol penetrated | 13999.3 | 14773.9 | 14603.7 | 11393.5 | 8292.1 |
|  | % Number fraction | 95.97 | 99.20 | 99.94 | 100 | 100 |
|  | Volume aerosol penetrated (µm^3^) | 1189.8 | 3856.4 | 17268.6 | 49956.7 | 63654.6 |
|  | % Volume fraction | 99.99 | 99.99 | 100 | 100 | 100 |
|  | Virion penetrated | 8406 | 27251 | 122029 | 353019 | 449815 |
| Fabric 3-layer | Number aerosol penetrated | 7937.4 | 7787.9 | 6546.2 | 3717.7 | 2056.9 |
|  | % Number fraction | 95.00 | 98.94 | 99.23 | 100 | 100 |
|  | Volume aerosol penetrated (µm^3^) | 483.00 | 1198.08 | 3058.61 | 4392.99 | 3724.54 |
|  | % Volume fraction | 99.98 | 99.99 | 100 | 100 | 100 |
|  | Virion penetrated | 3412 | 8466 | 21614 | 31043 | 26320 |
| Procedure | Number aerosol penetrated | 1720.7 | 1282.7 | 755.6 | 278.3 | 122.1 |
|  | % Number fraction | 86.31 | 96.25 | 99.64 | 99.98 | 100 |
|  | Volume aerosol penetrated (µm^3^) | 47.49 | 85.10 | 159.79 | 282.72 | 312.46 |
|  | % Volume fraction | 99.87 | 99.98 | 99.99 | 100 | 100 |
|  | Virion penetrated | 335 | 601 | 1129 | 1998 | 2208 |
| N95 | Number aerosol penetrated | 311.6 | 240.2 | 147.7 | 60.9 | 27.8 |
|  | % Number fraction | 89.91 | 97.26 | 99.74 | 99.99 | 100 |
|  | Volume aerosol penetrated (µm^3^) | 9.64 | 18.90 | 33.56 | 31.42 | 20.27 |
|  | % Volume fraction | 99.91 | 99.99 | 100 | 100 | 100 |
|  | Virion penetrated | 68 | 134 | 237 | 222 | 143 |

**S9 Table. The number and volume of aerosol, and the calculated virion penetrated, through the four mask group penetration profiles (see Figure 1 to 4) as a function of the number mean particle diameter for each challenge distribution, and a viral load concentration of 14.13 virion/um^3^.**

| **Mask type** |  | **Number mean diameter (µm) of challenge distribution** | | | | |
| --- | --- | --- | --- | --- | --- | --- |
|  |  | **0.3** | **0.5** | **1** | **2** | **3** |
| Fabric 2-layer | Number aerosol penetrated | 13999.3 | 14773.9 | 14603.7 | 11393.5 | 8292.1 |
|  | % Number fraction | 95.97 | 99.20 | 99.94 | 100 | 100 |
|  | Volume aerosol penetrated (µm^3^) | 1190.8 | 3858.2 | 17270.7 | 49956.7 | 63654.6 |
|  | % Volume fraction | 99.99 | 99.99 | 100 | 100 | 100 |
|  | Virion penetrated | 16827 | 54528 | 244087 | 706037 | 899630 |
| Fabric 3-layer | Number aerosol penetrated | 7937.4 | 7787.9 | 6546.2 | 3717.7 | 2056.9 |
|  | % Number fraction | 95.00 | 98.94 | 99.93 | 100 | 100 |
|  | Volume aerosol penetrated (µm^3^) | 483.49 | 1198.97 | 3059.61 | 4392.99 | 3724.54 |
|  | % Volume fraction | 99.98 | 100 | 100 | 100 | 100 |
|  | Virion penetrated | 6832 | 16945 | 43242 | 62086 | 52639 |
| Procedure | Number aerosol penetrated | 1720.7 | 1282.7 | 755.6 | 278.3 | 122.1 |
|  | % Number fraction | 86.31 | 96.25 | 99.94 | 99.98 | 100 |
|  | Volume aerosol penetrated (µm^3^) | 47.59 | 85.29 | 159.79 | 282.72 | 312.46 |
|  | % Volume fraction | 99.87 | 99.98 | 100 | 100 | 100 |
|  | Virion penetrated | 672 | 1205 | 2258 | 3996 | 4416 |
| N95 | Number aerosol penetrated | 311.6 | 240.2 | 147.7 | 60.9 | 27.8 |
|  | % Number fraction | 89.91 | 97.26 | 99.74 | 99.99 | 100 |
|  | Volume aerosol penetrated (µm^3^) | 9.64 | 18.90 | 33.56 | 31.42 | 20.27 |
|  | % Volume fraction | 99.91 | 99.99 | 100 | 100 | 100 |
|  | Virion penetrated | 136 | 267 | 474 | 444 | 287 |

**Conversion of mobility diameter to aerodynamic diameter**

Mobility particle diameter data obtained from the SMPS was converted to aerodynamic (equivalent) diameter and combined with the APS aerodynamic particle size data. Aerodynamic diameter finds wide application in aerosol technology as it standardizes a particle on shape (a sphere) and density (1g/cm^3^). The conversion of mobility to aerodynamic diameter is provided below:

$d_{a}=d_{m}\left[ \frac{C_{c}(d_{m})}{C_{c}(d_{a})} \right]^{\frac{1}{2}}\left( \frac{p_{p}}{\chi\rho_{o}} \right)^{\frac{1}{2}}$ S1 Eq.

Here ρ_p_ is the particle density, ρ_o_ the reference density 1 (grams per cm^3^) and χ is the dynamic shape factor. C_c_(d) is the slip correction:

$C_{c}=1+\frac{2\lambda}{D_{p}}\left( 1.142+.558e^{\frac{-.999D_{p}}{2\lambda}} \right)$ S2 Eq.

Where λ = Mean Free Path = 6.65e-8, and D_p_ is the diffusion coefficient for the particle diameter in question.

**Photographs of select masks evaluated**

Pictures are provided below of examples of the face coverings and respirators tested from each of the mask groups.


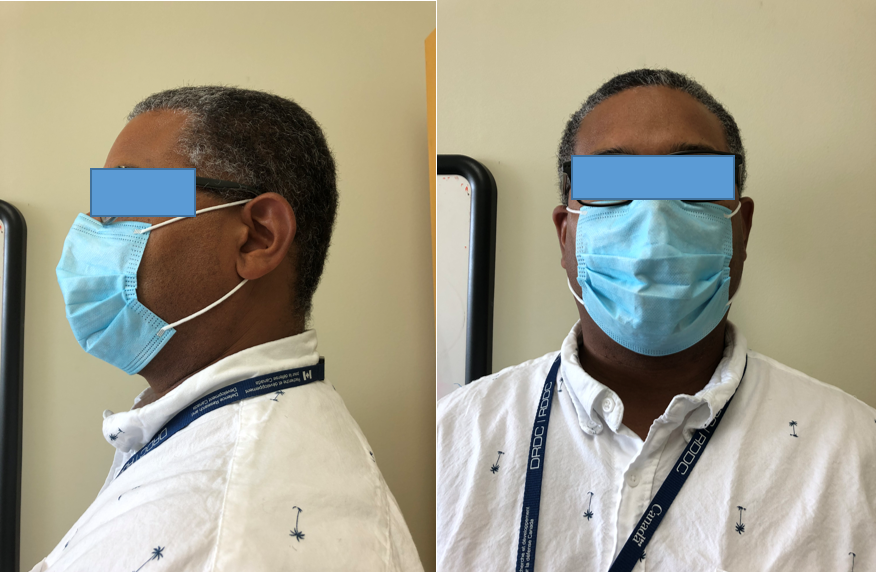


**S9 Fig. Disposable procedure mask (Henan Liwei), Henan Liwei Biological Pharmaceutical Co. Ltd.**


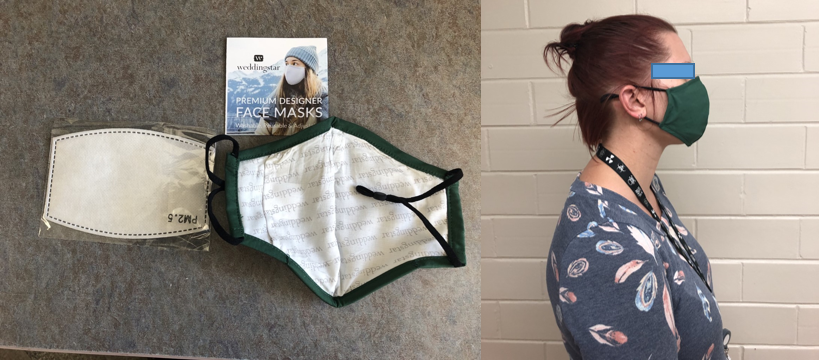


**S10 Fig. Multi-layer mask: 3-layer Cotton/PM2.5 filter insert/rayon (CFIR), Weddingstar (Medicine Hat, Canada).**


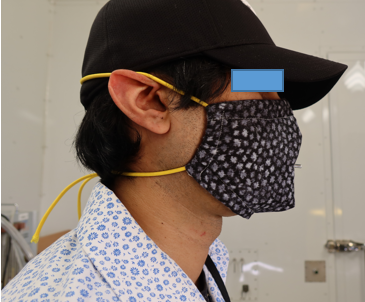


**S11 Fig. Multi-layer mask: four layered system comprised of two layers of quilt batting between
an outer and inner layer of cotton (C2QC).**


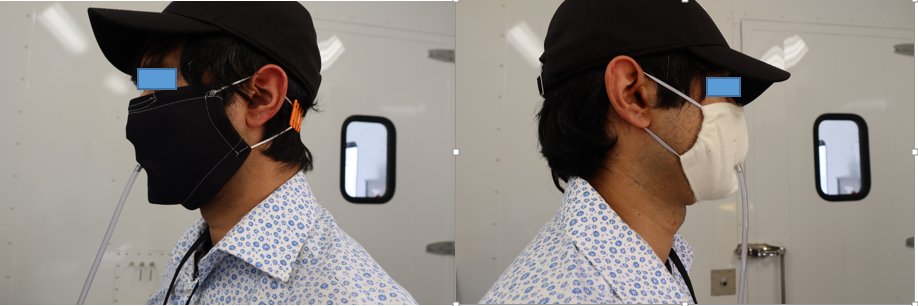


**S12 Fig. 2 Layer mask: silk fabric (SS, left) and Quilt batting/cotton mask (QC, right).**


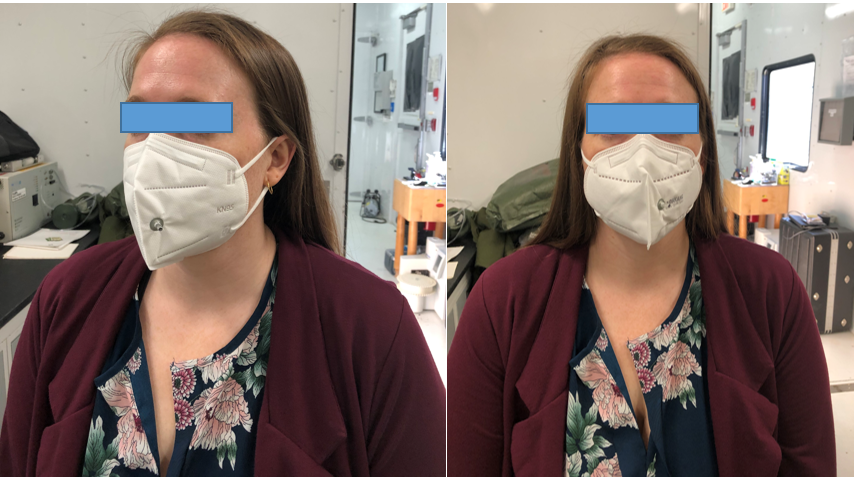


**S13 Fig. KN95 mask group: MedSup Canada Protective Face Mask (left) and TAIDAKANG Protective mask (right).**

**
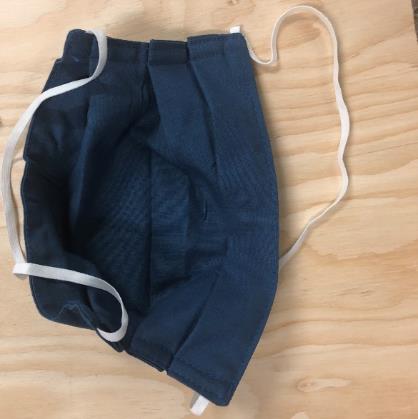
** **
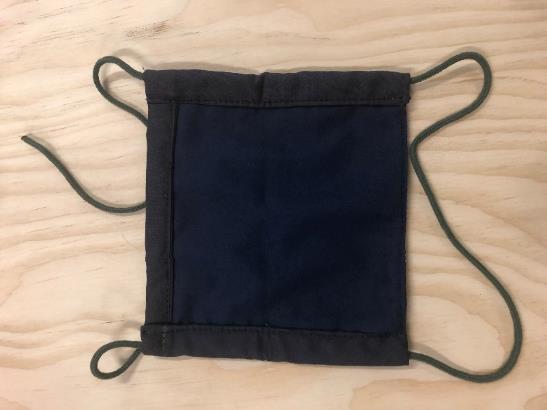
**

**S14 Fig. 2-layer mask group: cotton fabric variant 1 (CC, left) and cotton fabric variant 2 (right).**


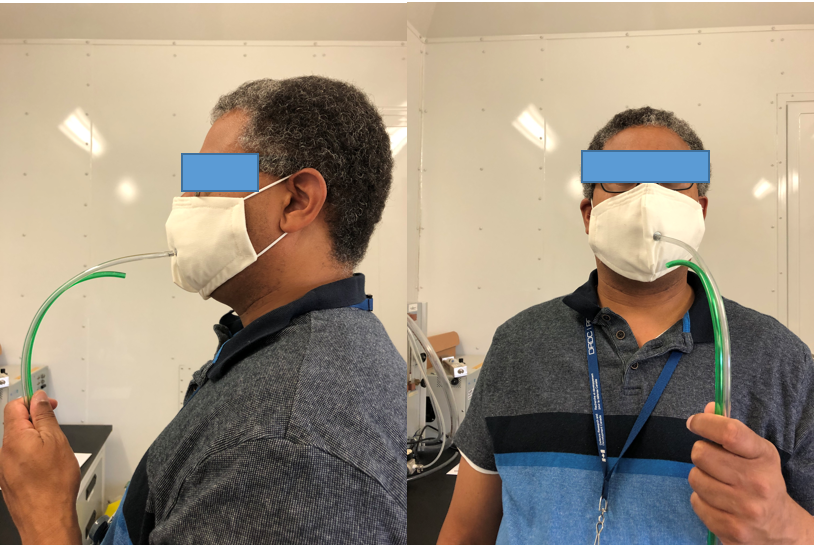


**S15 Fig. Multi-layer mask group: three layered system comprised of two layers of cotton between an inner layer of furnace filter (CFFC).**


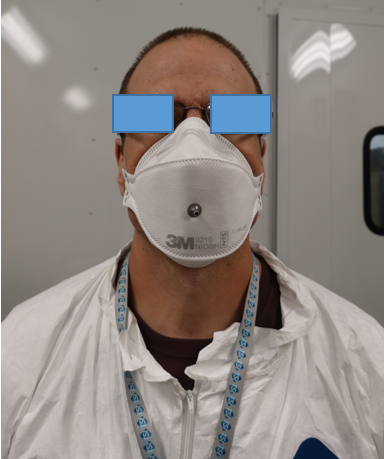


**S16 Fig. N95 FFR: 3M™ model** **9210.**


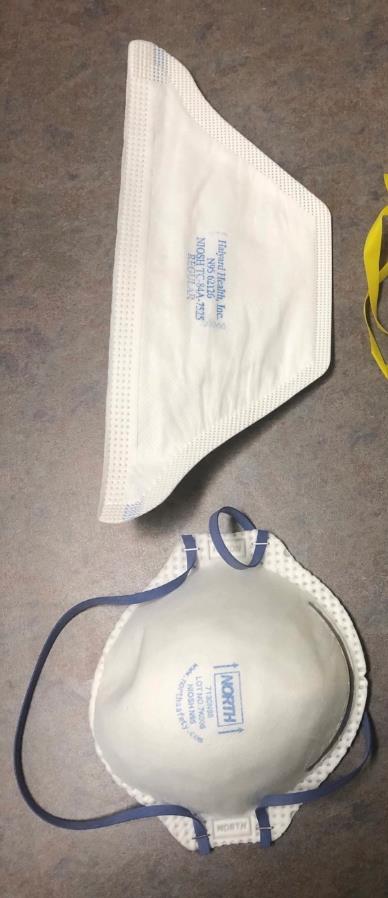


**S17 Fig. N95 FFR group: Halyard Health, model FLUIDSHIELD 2 N95 (duck bill fold style, left); North Safety Products model 7130N95 (cup style, right).**


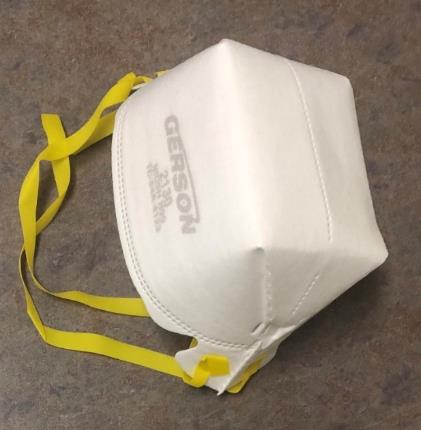
**
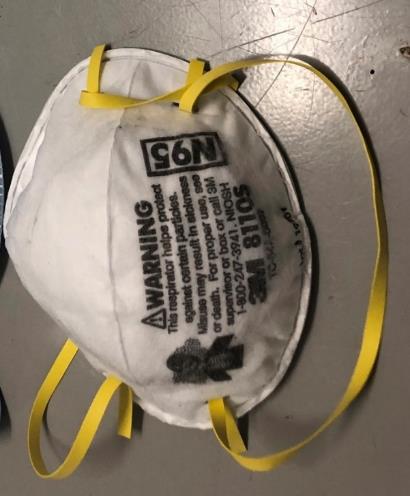
**
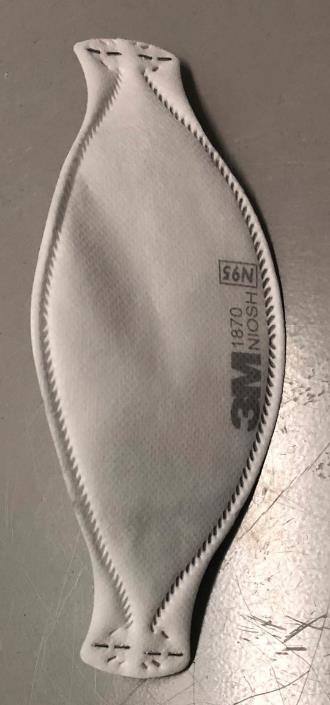


**S18 Fig. N95 FFR group: Gerson model 2130 N95 Respirator, Louis M. Gerson Co., (rectangular cup style, top left); 3M™ model 8110s (cup style, top right); 3M™ model 1870 (fold style, bottom);**

**
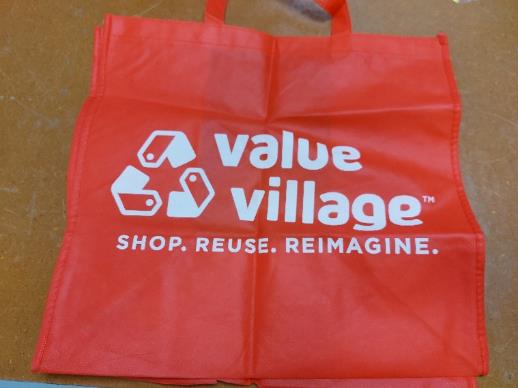

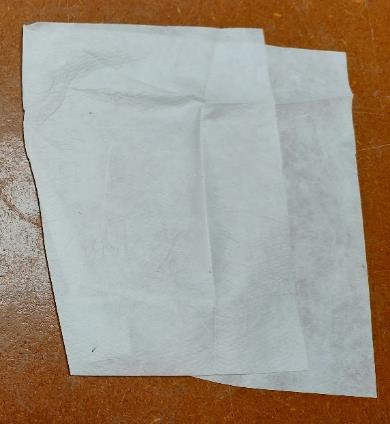
**

**S19 Fig. Non-woven polypropylene shopping bag (left) and “N95-like” electret filter membrane material (purchased from Amazon.ca) used as the middle filter layer, as part of the multi-layer mask group.**

**
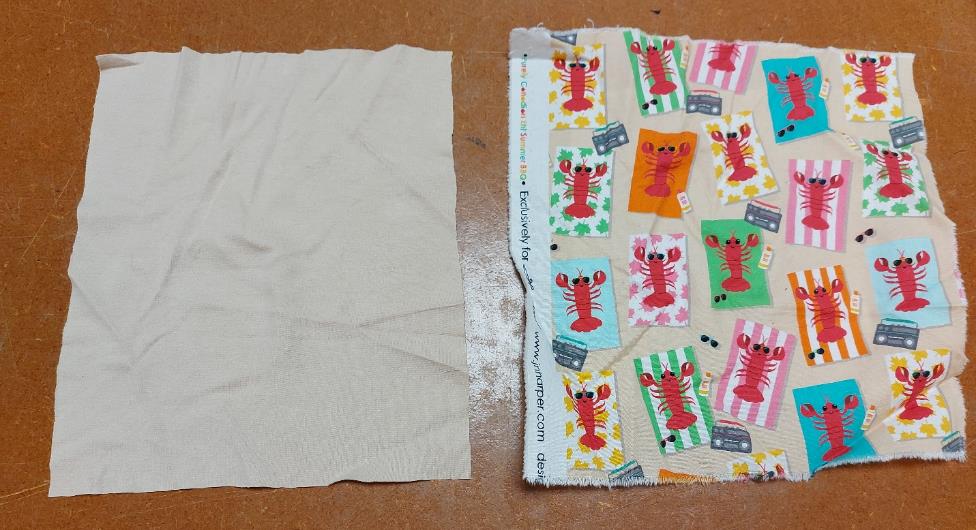
**

**S20 Fig. Quilt cotton (left) and cotton Knit material (right) used as an inner and outer layer, as part of the multi-layer mask group.**

**
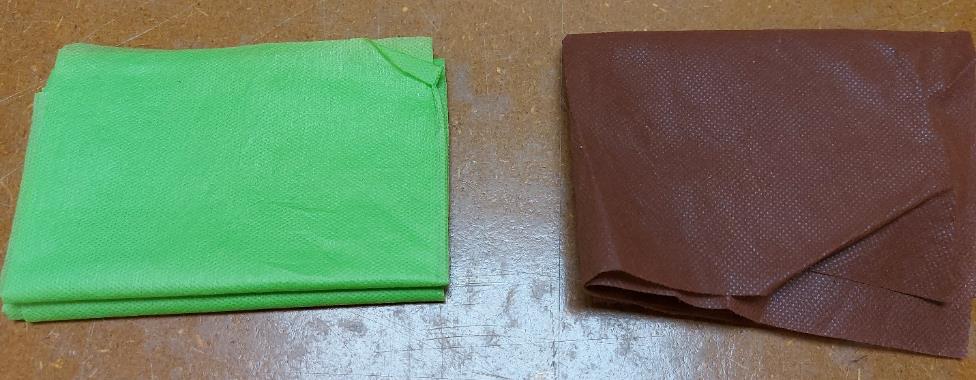
**

**S21. Fig. Non-woven polypropylene ‘craft’ material (manufactured by smart-fab®) used as a middle filter layer, as part of the multi-layer mask group.**

**Statistical analysis: total inward leakage**

The statistical analysis of the mean total inward leakage penetration and the mean fabric penetration data, for the five mask groups, are provide below. 2-L, M-L and PMs refer to the 2-layer, multi-layer and the disposable procedure mask group. Descriptive statistics, ANOVA one-way with a Bonholm test and a Shapiro-Wilk normality test was performed on the five mask groups.


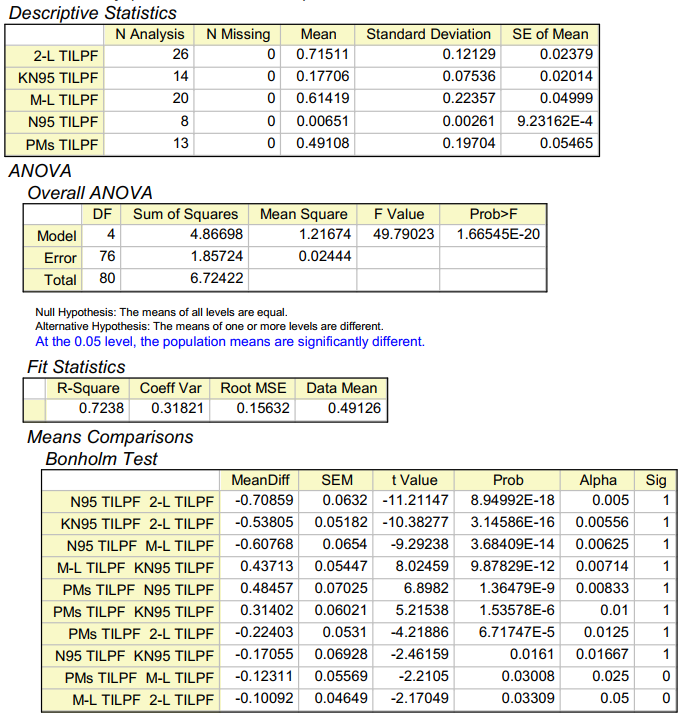


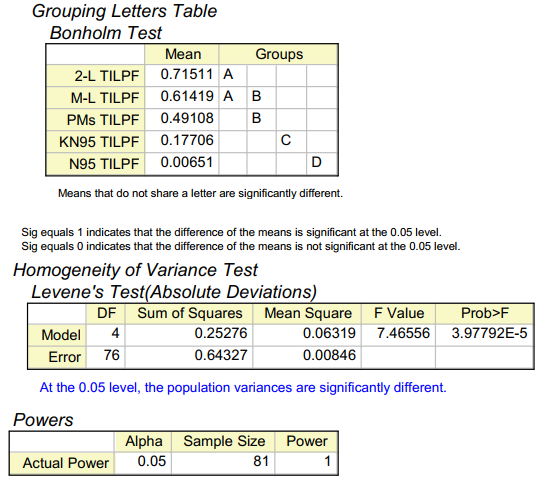


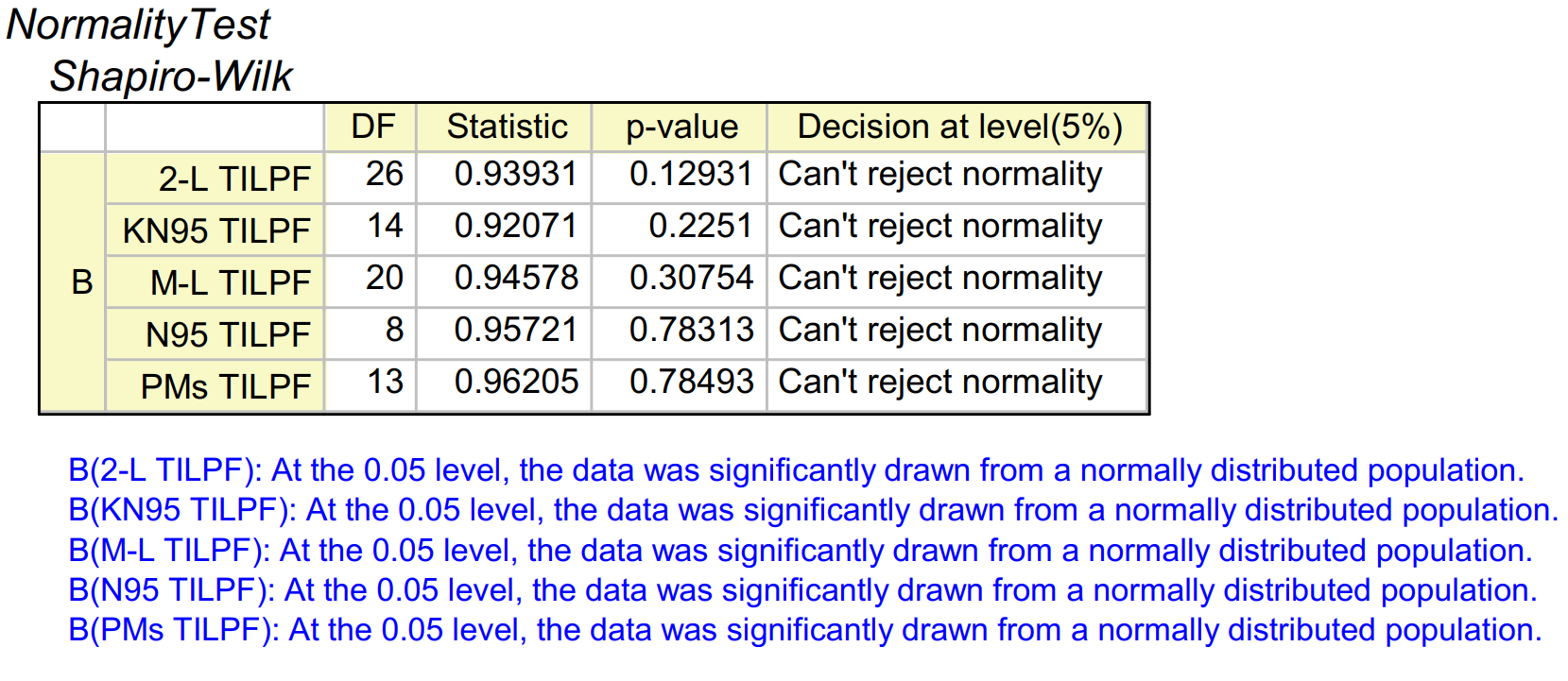


**S22 Fig. Descriptive statistics, ANOVA one-way with a Bonholm test and a Shapiro-Wilk normality test was performed on the mean total inward leakage penetration data for the five mask groups.**

**Statistical analysis: fabric penetration**

The statistical analysis of the mean fabric penetration data, for the five mask groups, are provide below. Kruskal-Wallis ANOVA and a Dunn’s Test was performed on two data sets of mask groups; 2-L, M-L and PMs mask groups, and the PM, KN95 and the N95 mask groups. A Shapiro-Wilk normality test was performed on the five mask groups.


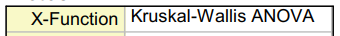


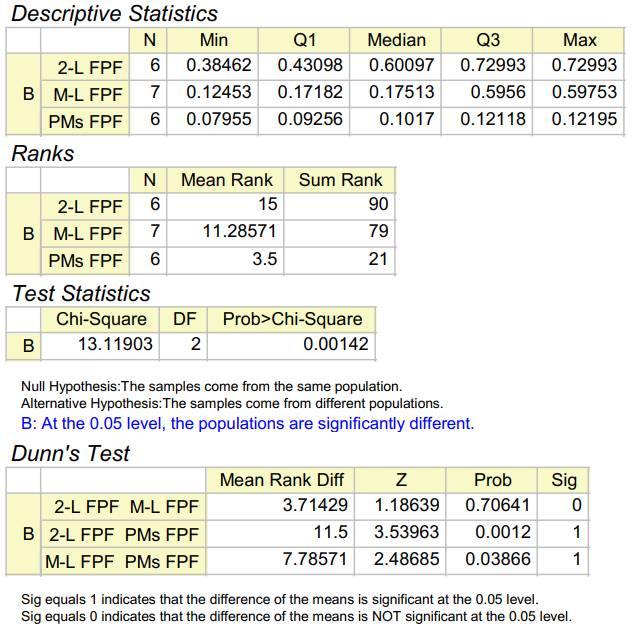


**S23 Fig. Descriptive statistics, Kruskal-Wallis ANOVA and Dunn’s test was performed on the mean fabric penetration data for the 2-L, M-L and PM mask group.**


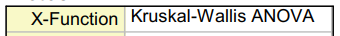


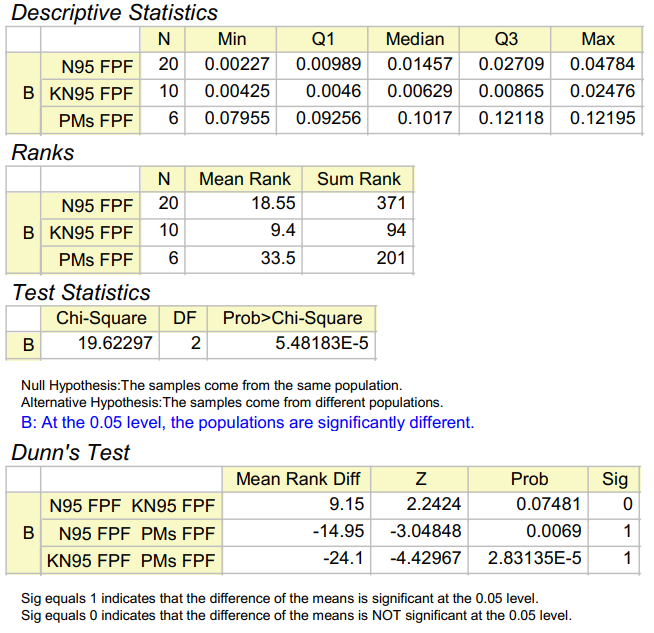


**S24 Fig. Descriptive statistics, Kruskal-Wallis ANOVA and Dunn’s test was performed on the mean fabric penetration data for the PM, N95 and KN95 mask group.**


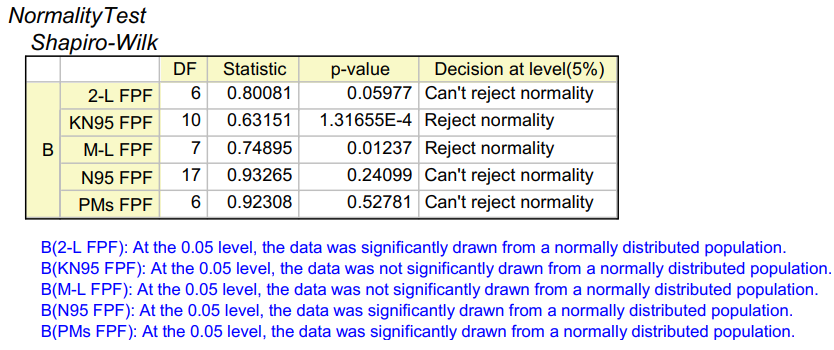


**S25 Fig. Shapiro-Wilk normality test was performed on the mean fabric penetration data for all five mask groups.**

**Supplementary References**

1. Asadi S, Wexler A, Cappa C, Barreda S, Bouvier N, & Ristenpart W. Aerosol emission and superemission during human speech increase with voice loudness. Nature; 2019 Feb. 9 p. Report No.: 2348.
2. Nicas M, Nazaroff WW, Hubbard A. Toward understanding the risk of secondary airborne infection: emission of respirable pathogens. J Occup Environ Hyg. 2005 Mar; 2(3): 143-154.
3. Mikhailov E, Vlasenko S, Niessner R, Poschl U. Interaction of aerosol particles composed of protein and salts with. Atmos Chem Phys. 2004 Feb; 4: 323-350.
4. Yang W, Marr L. Dynamics of airborne influenza A viruses indoors and dependence on humidity. PLoS One. 2011 Jun; 6(6): e21481.
5. Lee BU. Minimum sized of respiratory particles carrying SARS-CoV-2 and the possibility of aerosol generation. Int J Environ Res Public Health. 2020 Oct; 17(19): 6960.
6. Anand S, Mayya Y. Size distribution of virus laden droplets from expiratory ejecta of infected subjects. Nature; 2020 Dec. 10 p. Report No: 21174.
7. Hersen G, Stéphane M, Robine E, Géhin E, Corbet S, Vabert A, et al. Impact of Health on Particle Size of Exhaled Respiratory Aerosols: Case‐control Study. Clean (Weinh). 2008 July; 36(7): 572-577.
8. Riediker M, Tsai D-H. Estimation of viral aerosol emissions from simulated individuals with asymptomatic to moderate coronavirus disease 2019. JAMA Netw Open, 2020 Jul; 3(7): e2013807.
